# A characterization of finite abelian groups via sets of lengths in transfer Krull monoids

**DOI:** 10.1080/00927872.2018.1430811

**Published:** 2018-02-26

**Authors:** Qinghai Zhong

**Affiliations:** Institute for Mathematics and Scientific Computing, University of Graz, NAWI Graz, Graz, Austria

**Keywords:** Arithmetical characterizations, class groups, Davenport constant, Krull monoids, maximal orders, seminormal orders, sets of lengths, zero-sum sequences, 11B30, 11R27, 13A05, 13F05, 20M13

## Abstract

Let *H* be a transfer Krull monoid over a finite abelian group *G* (for example, rings of integers, holomorphy rings in algebraic function fields, and regular congruence monoids in these domains). Then each nonunit *a*∈*H* can be written as a product of irreducible elements, say $a=u_{1}\ldots u_{k}$, and the number of factors *k* is called the length of the factorization. The set *L*(*a*) of all possible factorization lengths is the set of lengths of *a*. It is classical that the system *ℒ*(*H*) = {*L*(*a*)∣*a*∈*H*} of all sets of lengths depends only on the group *G*, and a standing conjecture states that conversely the system *ℒ*(*H*) is characteristic for the group *G*. Let *H* ^*′*^ be a further transfer Krull monoid over a finite abelian group *G* ^*′*^ and suppose that *ℒ*(*H*) = *ℒ*(*H* ^*′*^). We prove that, if $G≅C_{n}^{r}$ with *r*≤*n*−3 or (*r*≥*n*−1≥2 and *n* is a prime power), then *G* and *G* ^*′*^ are isomorphic.

## 1. Introduction and main result

Let *H* be an atomic unit-cancellative monoid. Then each non-unit *a*∈*H* can be written as a product of atoms, and if $a=u_{1}\ldots u_{k}$ with atoms $u_{1},\ldots ,u_{k}$ of *H*, then *k* is called the length of the factorization. The set *L*(*a*) of all possible factorization lengths is the set of lengths of *a*, and *ℒ*(*H*) = {*L*(*a*)∣*a*∈*H*} is called the system of sets of lengths of *H* (for convenience we set *L*(*a*) = {0} if *a* is an invertible element of *H*). Under a variety of noetherian conditions on *H* (e.g., *H* is the monoid of nonzero elements of a commutative noetherian domain) all sets of lengths are finite. Sets of lengths (together with invariants controlling their structure, such as elasticities and sets of distances) are a well-studied means for describing the arithmetic structure of monoids.

Let *H* be a transfer Krull monoid over a finite abelian group *G*. Then, by definition, there is a weak transfer homomorphism *𝜃*:*H*→*ℬ*(*G*), where *ℬ*(*G*) denotes the monoid of zero-sum sequences over *G*, and hence *ℒ*(*H*) = *ℒ*(*ℬ*(*G*)). We use the abbreviation *ℒ*(*G*) = *ℒ*(*ℬ*(*G*)). By a result due to Carlitz in 1960, we know that *H* is half-factorial (i.e., |*L*| = 1 for all *L*∈*ℒ*(*H*)) if and only if |*G*|≤2. Suppose that |*G*|≥3. Then there is some *a*∈*H* with |*L*(*a*)|>1. If *k*,*ℓ*∈*L*(*a*) with *k*<*ℓ* and *m*∈*ℕ*, then *L*(*a* ^*m*^)⊃{*km*+*ν*(*ℓ*−*k*)∣*ν*∈[0,*m*]} which shows that sets of lengths can become arbitrarily large. Note that the system of sets of lengths of *H* depends only on the class group *G*. The associated inverse question asks whether or not sets of lengths are characteristic for the class group. In fact, we have the following conjecture (it was first stated in [6] and for a detailed description of the background of this problem, see [6], [7, Section 7.3], [8, page 42], and [24]).

Conjecture 1.1. Let *G* be a finite abelian group with *D*(*G*)≥4. If *G* ^*′*^ is an abelian group with *ℒ*(*G*) = *ℒ*(*G* ^*′*^), then *G* and *G* ^*′*^ are isomorphic.

Note if *D*(*G*) = 3, then we have $ℒ(C_{3})=ℒ(C_{2}⊕C_{2})$. The system of sets of lengths *ℒ*(*G*) is studied with methods from Additive Combinatorics. In particular, zero-sum theoretical invariants (such as the Davenport constant or the cross number) and the associated inverse problems play a crucial role. Most of these invariants are well-understood only in a very limited number of cases (e.g., for groups of rank two, the precise value of the Davenport constant *D*(*G*) is known and the associated inverse problem is solved; however, if *n* is not a prime power and *r*≥3, then the value of the Davenport constant $D(C_{n}^{r})$ is unknown). Thus it is not surprising that most aﬃrmative answers to the Characterization Problem so far have been restricted to those groups where we have a good understanding of the Davenport constant. These groups include elementary two-groups, cyclic groups, and groups of rank 2 (for recent progress we refer to [1, 9]).

The first and so far only groups, for which the Characterization Problem was solved whereas the Davenport constant is unknown, are groups of the form $C_{n}^{r}$, where *r*,*n*∈*ℕ* and 2*r*<*n*−2 (this is done by Geroldinger and Zhong [12] and Zhong [27]), which use a deep characterization of the structure of Δ*(*G*). In this paper, we go on to study groups of the form $C_{n}^{r}$ and obtain the following theorem.

Theorem 1.2. Let *H* be a transfer Krull monoid over a finite abelian group *G* with *D*(*G*)≥4. Suppose $G≅C_{n}^{r}$ with *r*,*n*∈*ℕ* and *ℒ*(*H*) = *ℒ*(*H* ^*′*^), where *H* ^*′*^ is a further transfer Krull monoid over a finite abelian group *G* ^*′*^. Then

1. If *r*≤*n*−3, then *G*≅*G* ^*′*^.
2. If *r*≥*n*−1 and *n* is a prime power, then *G*≅*G* ^*′*^.

This is made possible by introducing new invariants *ρ*(*G*,*d*) and ρ*(*G*,*d*) which are only depending on *ℒ*(*G*) (see Definitions 3.1 and 3.3). In Section 2 we gather the required background both on transfer Krull monoids as well as on Additive Combinatorics. In Section 3, we provide a detailed study of *ρ*(*G*,*d*) and ρ*(*G*,*d*). The proof of Theorem 1.2 will be provided in Section 4. The final section is concluding remarks and conjectures.

Throughout the paper, let $G≅C_{n_{1}}⊕\ldots ⊕C_{n_{r}}$ be a finite abelian group with *D*(*G*)≥4, where $r,n_{1},\ldots ,n_{r}\in \mathbb{N}$ and $1<n_{1}\;|\;\ldots \;|\;n_{r}$.

## 2. Background on transfer Krull monoids and sets of lengths

Our notation and terminology are consistent with [7, 9]. For convenience, we set min*∅* = 0. Let *ℕ* be the set of positive integers, let *ℤ* be the set of integers, let *ℚ* be the set of rational numbers, and let ℝ be the set of real numbers. For rational numbers *a*,*b*∈*ℚ*, we denote by [*a*,*b*] = {*x*∈*ℤ*∣*a*≤*x*≤*b*} the discrete, finite interval between *a* and *b*. If *L*⊂*ℕ* is a subset, then *Δ*(*L*) denotes the set of (successive) distances of *L* (that is, *d*∈*Δ*(*L*) if and only if *d* = *b*−*a* with *a*,*b*∈*L* distinct and [*a*,*b*]∩*L* = {*a*,*b*}) and *ρ*(*L*) = sup*L*∕min*L* denotes its elasticity (for convenience, we set *ρ*({0}) = 1).

Let *r*∈*ℕ* and let $\left(e_{1},\ldots ,e_{r}\right)$ be an *r*-tuple of elements of *G*. Then $\left(e_{1},\ldots ,e_{r}\right)$ is said to be independent if *e*_*i*_≠0 for all *i*∈[1,*r*] and if for all $(m_{1},\ldots ,m_{r})\in {\mathbb{Z}}^{r}$ an equation $m_{1}e_{1}+\ldots +m_{r}e_{r}=0$ implies that $m_{i}e_{i}=0$ for all *i*∈[1,*r*]. Furthermore, $\left(e_{1},\ldots ,e_{r}\right)$ is said to be a basis of *G* if it is independent and $G=\langle e_{1}\rangle ⊕\ldots ⊕\langle e_{r}\rangle$. For every *n*∈*ℕ*, we denote by *C*_*n*_ an additive cyclic group of order *n*. Since $G≅C_{n_{1}}⊕\ldots ⊕C_{n_{r}}$, *r* = *r*(*G*) is the rank of *G* and *n*_*r*_ = exp(*G*) is the exponent of *G*.

### 2.1. Sets of lengths

By a monoid, we mean an associative semigroup with unit element, and if not stated otherwise we use multiplicative notation. Let *H* be a monoid with unit element 1 = 1_*H*_∈*H*. An element *a*∈*H* is said to be invertible (or an unit) if there exists an element *a* ^*′*^∈*H* such that $aa^{\prime }=a^{\prime }a=1$. The set of invertible elements of *H* will be denoted by *H* ^×^, and we say that *H* is reduced if *H* ^×^ = {1}. The monoid *H* is said to be unit-cancellative if for any two elements *a*,*u*∈*H*, each of the equations *au* = *a* or *ua* = *a* implies that *u*∈*H* ^×^. Clearly, every cancellative monoid is unit-cancellative.

Suppose that *H* is unit-cancellative. An element *u*∈*H* is said to be irreducible (or an atom) if *u*∉*H* ^×^ and for any two elements *a*,*b*∈*H*, *u* = *ab* implies that *a*∈*H* ^×^ or *b*∈*H* ^×^. Let *𝒜*(*H*) denote the set of atoms, and we say that *H* is atomic if every non-unit is a finite product of atoms. If *H* satisfies the ascending chain condition on principal left ideals and on principal right ideals, then *H* is atomic [4, Theorem 2.6]. If *a*∈*H*∖*H* ^×^ and $a=u_{1}\ldots u_{k}$, where *k*∈*ℕ* and $u_{1},\ldots ,u_{k}\in 𝒜(H)$, then *k* is a factorization length of *a*, and

$$\begin{array}{c} L_{H}(a)=L(a)=\{k|k\;\text{is a factorization length of}\;a\}\subset \mathbb{N} \end{array}$$

denotes the set of lengths of *a*. It is convenient to set *L*(*a*) = {0} for all *a*∈*H* ^×^. The family

$$\begin{array}{c} ℒ(H)=\{L(a)|a\in H\} \end{array}$$

is called the system of sets of lengths of *H*, and

$$\begin{array}{c} \rho (H)=\sup\{\rho (L)|L\in ℒ(H)\}\in {\mathbb{R}}_{\geq 1}\cup \{\infty \} \end{array}$$

denotes the elasticity of *H*. We call

$$\begin{array}{c} \Delta (H)=\underset{L\in ℒ(H)}{⋃}\Delta (L)\;\subset \mathbb{N} \end{array}$$

the set of distances of *H*. Note that *Δ*(*H*) can be infinite, and by definition we have *Δ*(*H*) = *∅* if and only if *ρ*(*H*) = 1. If *Δ*(*H*)≠*∅*, then we have min*Δ*(*H*) = gcd*Δ*(*H*) [3, Proposition 2.9].

### 2.2. Monoids of zero-sum sequences

Let *G*_0_⊂*G* be a non-empty subset. Then ⟨*G*_0_⟩ denotes the subgroup generated by *G*_0_. In Additive Combinatorics, a sequence (over *G*_0_) means a finite sequence of terms from *G*_0_ where repetition is allowed and the order of the elements is disregarded, and (as usual) we consider sequences as elements of the free abelian monoid with basis *G*_0_. Let

$$\begin{array}{c} S=g_{1}\ldots g_{\ell }=\underset{g\in G_{0}}{\prod }g^{v_{g}(S)}\in ℱ(G_{0}) \end{array}$$

be a sequence over *G*_0_. We call

$$\begin{array}{ccc} supp(S) & = & \{g\in G|v_{g}(S)>0\}\subset G\;\text{the}\;support\;\text{of }S\;,\\ \left|S\right| & = & \ell =\underset{g\in G}{\sum }v_{g}(S)\in {\mathbb{N}}_{0}\;\text{the}\;length\;\text{of }S\;,\\ \sigma (S) & = & \overset{\ell }{\underset{i=1}{\sum }}g_{i}\;\text{the}\;sum\;\text{of }S\;,\\ \;\;\text{ and }\;\;\;\Sigma (S) & = & \{\underset{i\in I}{\sum }g_{i}|\emptyset \neq I\subset [1,\ell ]\}\;\text{ the}\;setofsubsequencesums\;\text{of }S\;. \end{array}$$

The sequence *S* is said to be

- *zero-sum free* if 0∉*Σ*(*S*),
- a *zero-sum sequence* if *σ*(*S*) = 0,
- a *minimal zero-sum sequence* if it is a nontrivial zero-sum sequence and every proper subsequence is zero-sum free.

The set of zero-sum sequences $ℬ(G_{0})=\{S\in ℱ(G_{0})|\sigma (S)=0\}\subset ℱ(G_{0})$ is a submonoid, and the set of minimal zero-sum sequences is the set of atoms of *ℬ*(*G*_0_). For any arithmetical invariant ∗(*H*) defined for a monoid *H*, we write ∗(*G*_0_) instead of ∗(*ℬ*(*G*_0_)). In particular, $𝒜(G_{0})=𝒜(ℬ(G_{0}))$ is the set of atoms of *ℬ*(*G*_0_), $ℒ(G_{0})=ℒ(B(G_{0}))$ is the system of sets of lengths of *ℬ*(*G*_0_), and so on. We denote by

$$\begin{array}{c} D(G_{0})=\max\{|S||S\in 𝒜(G_{0})\}\in \mathbb{N} \end{array}$$

the Davenport constant of *G*_0_.

### 2.3. Transfer Krull monoids

Let *H* be a atomic unit-cancellative monoid.

1. We say a monoid homomorphism *𝜃*:*H*→*B* to an atomic unit-cancellative monoid *B* is a weak transfer homomorphism if it has the following two properties:
  - $B=B^{\times }𝜃(H)B^{\times }$ and ${𝜃}^{-1}(B^{\times })=H^{\times }$.
  - If *a*∈*H*, *n*∈*ℕ*, $v_{1},\ldots ,v_{n}\in 𝒜(B)$ and $𝜃(a)=v_{1}\ldots v_{n}$, then there exist $u_{1},\ldots ,u_{n}\in 𝒜(H)$ and a permutation *τ*∈*𝔖*_*n*_ such that $a=u_{1}\ldots u_{n}$ and $𝜃(u_{i})\in B^{\times }v_{\tau (i)}B^{\times }$ for each *i*∈[1,*n*].
  Let *𝜃*:*H*→*B* be a weak transfer homomorphism between atomic unit-cancellative monoids. It follows that for all *a*∈*H*, we have $L_{H}(a)=L_{B}(𝜃(a))$ and hence *ℒ*(*H*) = *ℒ*(*B*).
2. We say *H* is a *transfer Krull monoid* if one of the following equivalent conditions holds:
  - There exists a weak transfer homomorphism *𝜃*:*H*→ℋ* for a commutative Krull monoid ℋ* (i.e., ℋ* is commutative, cancellative, completely integrally closed, and *v*-noetherian).
  - There exists a weak transfer homomorphism ${𝜃}^{\prime }:H\to ℬ(G_{0})$ for a subset *G*_0_ of an abelian group.
  If the second condition holds, then we say *H* is a transfer Krull monoid over *G*_0_. If *G*_0_ is finite, then *H* is said to be a *transfer Krull monoid of finite type*.
3. We say a domain *R* is a *transfer Krull domain* (of finite type) if its monoid of cancelative elements *R* ^∙^ is a transfer Krull monoid (of finite type).

In particular, commutative Krull monoids are transfer Krull monoids. Rings of integers, holomorphy rings in algebraic function fields, and regular congruence monoids in these domains are commutative Krull monoids with finite class group such that every class contains a prime divisor [7, Section 2.11 and Examples 7.4.2]. Monoid domains and power series domains that are Krull are discussed in [17, 2], and note that every class of a Krull monoid domain contains a prime divisor. Thus all these commutative Krull monoids are transfer Krull monoids over a finite abelian group.

However, a transfer Krull monoid need neither be commutative nor *v*-noetherian nor completely integrally closed. To give a noncommutative example, let *𝒪* be a holomorphy ring in a global field *K*, *A* a central simple algebra over *K*, and *H* a classical maximal *𝒪*-order of *A* such that every stably free left *R*-ideal is free. Then *H* is a transfer Krull monoid over a ray class group of *𝒪* [25, Theorem 1.1]. Let *R* be a bounded HNP (hereditary noetherian prime) ring. If every stably free left *R*-ideal is free, then its multiplicative monoid of cancelative elements is a transfer Krull monoid [26, Theorem 4.4]. A class of commutative weakly Krull domains which are transfer Krull but not Krull will be given in [10, Theorem 5.8]. Extended lists of commutative Krull monoids and of transfer Krull monoids, which are not commutative Krull, are given in [6].

Let *G*_0_⊂*G* be a non-empty subset. For a sequence $S=g_{1}\ldots g_{\ell }\in ℱ(G_{0})$, we call

$$\begin{array}{ccc} k(S) & = & \overset{l}{\underset{i=1}{\sum }}\frac{1}{ord(g_{i})}\;\in {\mathbb{Q}}_{\geq 0}\;\text{the}crossnumber\text{of}\;S\text{, and }\\ K(G_{0}) & = & \max\{k(S)|S\in 𝒜(G_{0})\}\;\text{the}crossnumber\text{of}G_{0}. \end{array}$$

They were introduced by U. Krause in 1984 (see [19]) and were studied under various aspects. For the relevance with the theory of non-unique factorizations, see [21, 20, 22, 23] and [7, Chapter 6].

Suppose $G≅C_{q_{1}}⊕\ldots ⊕C_{q_{r^{*}}}$, where r* is the total rank of *G* and $q_{1},\ldots ,q_{r^{*}}$ are prime powers, and set

$$\begin{array}{c} K^{*}(G)=\frac{1}{\exp(G)}+\overset{r^{*}}{\underset{i=1}{\sum }}\frac{q_{i}-1}{q_{i}}\;. \end{array}$$

It is easy to see that K*(*G*)≤*K*(*G*) and there is known no group for which inequality holds. For further progress on *K*(*G*), we refer to [5, 15, 16, 18].

Lemma 2.1. If *G* is a *p*-group, then *K*(*G*) = K*(*G*)<*r*(*G*).

Proof. See [7, Theorem 5.5.9].

A subset *G*_0_⊂*G* is called half-factorial if *Δ*(*G*_0_) = *∅*. Otherwise, *G*_0_ is called non-half-factorial. Furthermore, the set *G*_0_ is called

- minimal non-half-factorial if it is non-half-factorial and every proper subset $G_{1}⊊G_{0}$ is half-factorial.
- an LCN-set if *k*(*A*)≥1 for all *A*∈*𝒜*(*G*_0_).

We collect some easy or well known results which will be used throughout the manuscript without further mention.

Lemma 2.2. Let *G*_0_⊂*G* be a non-empty subset. Then

1. *G*_0_ is half-factorial if and only if *k*(*A*) = 1 for all *A*∈*𝒜*(*G*_0_).
2. If *G*_0_ is an LCN-set, then $\min\Delta (G_{0})\leq |G_{0}|-2$.
3. *ℬ*(*G*_0_) has accepted elasticity.
4. $\rho (G)=\frac{D(G)}{2}$.
5. *Δ*(*G*) is an interval with min*Δ*(*G*) = 1.
6. If *B*∈*ℬ*(*G*), then *ρ*(*L*(*B* ^*k*^))≥*ρ*(*L*(*B*)) for every *k*∈*ℕ*.
7. If *A*∈*𝒜*(*G*), then {exp(*G*),exp(*G*)*k*(*A*)}⊂*L*(*A* ^exp(*G*)^).

Proof. 1. follows from [7, Proposition 6.7.3] and 2. from [7, Lemma 6.8.6].

2. see [7, Proposition 6.7.3, Lemma 6.8.6].

3. follows from [7, Theorem 3.1.4] and 4. from [7, Section 6.3].

4. See [7, Theorem 3.1.4, Section 6.3].

5. See [11].

6. Let *B*∈*ℬ*(*G*) and *k*∈*ℕ*. Then max*L*(*B* ^*k*^)≥*k*max*L*(*B*) and min*L*(*B* ^*k*^)≤*k*min*L*(*B*). It follows that $\rho (L(B^{k}))=\frac{\max L(B^{k})}{\min L(B^{k})}\geq \frac{k\max L(B)}{k\min L(B)}=\rho (L(B))$.

7. Let *A*∈*𝒜*(*G*) and suppose $A=g_{1}\ldots g_{\ell }$, where *ℓ*∈*ℕ* and $g_{1},\ldots ,g_{\ell }\in G$. Then

$$\begin{array}{c} A^{\exp(G)}=\overset{\ell }{\underset{i=1}{\prod }}{\left(g_{i}^{ord(g_{i})}\right)}^{\frac{\exp(G)}{ord(g_{i})}}\;. \end{array}$$

Since *A*, $g_{1}^{ord(g_{1})},\ldots ,g_{\ell }^{ord(g_{\ell })}$ are atoms, we obtain {exp(*G*),exp(*G*)*k*(*A*)}⊂*L*(*A* ^exp(*G*)^).

We need the following lemma.

Lemma 2.3. Let $e_{1},\ldots ,e_{r}\in G$ be independent elements with the same order *n*, where *r*, *n*∈*ℕ*_≥2_. Then

1.
  $$\begin{array}{c} \Delta (\{e_{1}+\ldots +e_{r},e_{1},\ldots ,e_{r}\})=\{r-1\}\;. \end{array}$$
2. If *n*≠*r*+1, then
  $$\begin{array}{c} \Delta (\{-(e_{1}+\ldots +e_{r}),e_{1},\ldots ,e_{r}\})=\{|n-r-1|\}\;. \end{array}$$
3. If *n* = *r*+1, then
  $$\begin{array}{c} \min\Delta (\{-(e_{1}+\ldots +e_{r}),e_{1}+\ldots +e_{r},e_{1},\ldots ,e_{r}\})=r-1\;. \end{array}$$

Proof. 1. See [7, Proposition 6.8.2].

2. See [7, Proposition 4.1.2.5].

3. Let $g=e_{1}+\ldots +e_{r}$ and $G_{0}=\{g,-g,e_{1},\ldots ,e_{r}\}$. Let *B*∈*ℬ*(*G*_0_) and assume

$$\begin{array}{c} B=U_{1}\ldots U_{k}=V_{1}\ldots V_{\ell },\text{ where }k,\ell \in N\text{ and }U_{1},\ldots ,U_{k},V_{1},\ldots ,V_{\ell }\in 𝒜(G_{0})\;. \end{array}$$

Let

$$\begin{array}{ccc} I_{1} & = & \left\{i\in [1,k]|v_{g}(U_{i})>0\text{ and }v_{-g}(U_{i})=0\},\;\;\;I_{2}=\{i\in [1,k]|U_{i}=g(-g)\right\}\\ J_{1} & = & \{j\in [1,\ell ]|v_{g}(V_{j})>0\text{ and }v_{-g}(V_{j})=0\},\;\;\;J_{2}=\{j\in [1,\ell ]|V_{j}=g(-g)\}\;. \end{array}$$

Then for every *i*∈*I*_1_, $k(U_{i})=1+\frac{n-v_{g}(U_{i})}{n}(n-2)$ and for every *j*∈*J*_1_, $k(V_{j})=1+\frac{n-v_{g}(V_{j})}{n}(n-2)$.

Note that for every $i\in [1,k]\setminus (I_{1}\cup I_{2})$ and every $j\in [1,\ell ]\setminus (J_{1}\cup J_{2})$, $k(U_{i})=k(V_{j})=1$. Therefore

$$\begin{array}{c} k(B)=k-|I_{1}|-|I_{2}|+\underset{i\in I_{1}}{\sum }(1+\frac{\left(n-v_{g}(U_{i}))(n-2\right)}{n})+\underset{i\in I_{2}}{\sum }\frac{2}{n}=k+(n-2)|I_{1}|-\frac{n-2}{n}v_{g}(B) \end{array}$$

and

$$\begin{array}{c} k(B)=\ell -|J_{1}|-|J_{2}|+\underset{j\in J_{1}}{\sum }(1+\frac{\left(n-v_{g}(V_{j}))(n-2\right)}{n})+\underset{j\in J_{2}}{\sum }\frac{2}{n}=\ell +(n-2)|J_{1}|-\frac{n-2}{n}v_{g}(B)\;. \end{array}$$

It follows that $k-\ell =(n-2)(|J_{1}|-|I_{1}|)$ and hence *n*−2 | min*Δ*(*G*_0_). By 1., we obtain *n*−2 = *r*−1 = min*Δ*(*G*_0_).

If *d*∈*ℕ* and *ℓ*,*M*∈*ℕ*_0_, then a finite subset *L*⊂*ℤ* is called an almost arithmetical progression (AAP for short) with difference *d*, length *ℓ*, and bound *M* if

(1) $$\begin{array}{c} L=y+(L^{\prime }\cup \{0,d,\ldots ,\ell d\}\cup {L{}^{\prime }}^{\prime })\subset y+d\mathbb{Z} \end{array}$$

where *y*∈*ℤ*, *L* ^*′*^⊂[−*M*,−1], and ${L{}^{\prime }}^{\prime }\subset \ell d+[1,M]$.

Lemma 2.4. There exist constants $M_{1},M_{2}\in \mathbb{N}$ such that for every *A*∈*ℬ*(*G*) with *Δ*(*supp*(*A*))≠*∅*, the set $L(A^{M_{1}})$ is an AAP with difference min*Δ*(*supp*(*A*)), length at least 1, and bound *M*_2_.

Proof. See [7, Theorem 4.3.6].

Next, we recall the definition of the invariants Δ*(*G*) and *Δ*_1_(*G*) (see [7, Definition 4.3.12]) in the Characterization Problem.

Let

$$\begin{array}{c} \Delta^{*}(G)=\{\min\Delta (G_{0})|G_{0}\subset G\text{ is a subset with }\Delta (G_{0})\neq \emptyset \}\;. \end{array}$$

We define

$$\begin{array}{ccc} m(G) & = & \max\{\min\Delta (G_{0})|G_{0}\subset G\;\text{is an LCN-set with}\;\Delta (G_{0})\neq \emptyset \}\;, \end{array}$$

and we denote by *Δ*_1_(*G*) the set of all *d*∈*ℕ* with the following property:

- For every *k*∈*ℕ*, there exists some *L*∈*ℒ*(*G*) which is an AAP with difference *d* and length *ℓ*≥*k*.

Lemma 2.5. Let *k*∈*ℕ* be maximal such that *G* has a subgroup isomorphic to $C_{\exp(G)}^{k}$. Then

1. $\Delta^{*}(G)\subset \Delta_{1}(G)\subset \{d\in \Delta (G)|\text{there exists }d^{\prime }\in \Delta^{*}(G)\text{ such that }d\;|\;d^{\prime }\}$.
2. $\max\Delta^{*}(G)=\max\Delta_{1}(G)=\max\{\exp(G)-2,r(G)-1\}$.
3. $m(G)\leq \max\{r(G)-1,\lfloor \frac{\exp(G)}{2}\rfloor -1\}$.
4. $\left\{1,r(G)-1,\exp(G)-2\}\subset \Delta^{*}(G)\subset \Delta_{1}(G)\subset [1,\max\{r(G)-1,\lfloor \frac{\exp(G)}{2}\rfloor -1\}]\cup [\max\{1,\exp(G)-k-1,\exp(G)-2\}\right]$.
5. *Δ*_1_(*G*) is an interval if and only if Δ*(*G*) is an interval if and only if *r*(*G*)+*k*≥exp(*G*)−1 or $G≅C_{2r(G)+2}^{r(G)}$.

Proof. 1. Follows from [7, Corollary 4.3.16]

2. From [14, Theorem 1.1].

3. See [27, Proposition 3.7].

4. Follows from 1. and [27, Theorem 1.1].

5. If *Δ*_1_(*G*) is an interval, then $\exp(G)-k-2\leq \max\{r(G)-1,\lfloor \frac{\exp(G)}{2}\rfloor -1\}$ by 4 which implies that Δ*(*G*) is an interval by Zhong [27, Theorem 1.1.2]. If Δ*(*G*) is an interval, then *Δ*_1_(*G*) is an interval by 1.. It follows by Zhong [27, Theorem 1.1.2] that Δ*(*G*) is an interval if and only if *r*(*G*)+*k*≥exp(*G*)−1 or $G≅C_{2r(G)+2}^{r(G)}$.

Lemma 2.6. Let *G*_0_⊂*G* with min*Δ*(*G*_0_)≥⌊exp(*G*)∕2⌋. Then

1. Let *A*∈*𝒜*(*G*_0_) with *k*(*A*)<1. Then $k(A)=\frac{\exp(G)-\min\Delta (G_{0})}{\exp(G)}$ and *ord*(*h*) = exp(*G*), *v*_*h*_(*A*) = 1 for all *h*∈*supp*(*A*).
2. Let *A*∈*𝒜*(*G*_0_) with *k*(*A*)≥1. Then *supp*(*A*) is an LCN-set and
  $$\begin{array}{c} \min\Delta (supp(A))\leq |supp(A)|-2\;. \end{array}$$
3. If *G*_0_ is a minimal non-half-factorial LCN-set with $\min\Delta (G_{0})=\max\Delta^{*}(G)$, then |*G*_0_| = *r*(*G*)+1 and for every *h*∈*G*_0_, we have *r*(⟨*G*_0_∖{*h*}⟩) = *r*(*G*).
4. Let *B*∈*ℬ*(*G*) with *ρ*(*L*(*B*)) = *ρ*(*G*). Suppose $B=U_{1}\ldots U_{k}=V_{1}\ldots V_{\ell }$, where *k* = min*L*(*B*), *ℓ* = max*L*(*B*), and $U_{1},\ldots ,U_{k},V_{1},\ldots V_{\ell }$ are atoms. Then *k*(*U*_*i*_)≥1 for all *i*∈[1,*k*] and *k*(*V*_*j*_)≤1 for all *j*∈[1,*ℓ*].

Proof. 1. Since {exp(*G*),exp(*G*)*k*(*A*)}⊂*L*(*A* ^exp(*G*)^), we have

$$\begin{array}{c} \min\Delta (supp(A))\;|\;\exp(G)-\exp(G)k(A) \end{array}$$

and hence

$$\begin{array}{c} \min\Delta (supp(A))\leq \exp(G)-\exp(G)k(A)\leq \exp(G)-2\;. \end{array}$$

Since min*Δ*(*G*_0_) | min*Δ*(*supp*(*A*)) and min*Δ*(*G*_0_)≥⌊exp(*G*)∕2⌋, it follows that

$$\begin{array}{ccc} \frac{1}{2}(\exp(G)-2) & < & \left\lfloor \exp(G)∕2\rfloor \leq \min\Delta (G_{0})\leq \min\Delta (supp(A)\right)\\ \leq & \exp(G)-\exp(G)k(A)\leq \exp(G)-2\;. \end{array}$$

Therefore min*Δ*(*G*_0_) = min*Δ*(*supp*(*A*)) = exp(*G*)−exp(*G*)*k*(*A*) and hence $k(A)=\frac{\exp(G)-\min\Delta (G_{0})}{\exp(G)}$.

Let *g*∈*supp*(*A*) such that $\frac{ord(g)}{v_{g}(A)}=\min\{\frac{ord(h)}{v_{h}(A)}|h\in supp(A)\}$. Then $A^{\left\lceil \frac{ord(g)}{v_{g}(A)}\right\rceil }=g^{ord(g)}\cdot B_{1}$, where *B*_1_∈*ℬ*(*supp*(*A*)). If there exists an atom *A*_1_ with *k*(*A*_1_)<1 such that *A*_1_ divides *B*_1_, then $k(A_{1})=\frac{\exp(G)-\min\Delta (G_{0})}{\exp(G)}=k(A)$. Therefore $1+\max L(B_{1})<\lceil \frac{ord(g)}{v_{g}(A)}\rceil$ and hence $\lfloor \exp(G)∕2\rfloor \leq \min\Delta (G_{0})\leq \lceil \frac{ord(g)}{v_{g}(A)}\rceil -2$. It follows by the minimality of $\frac{ord(g)}{v_{g}(A)}$ that *ord*(*h*) = exp(*G*) and *v*_*h*_(*A*) = 1 for all *h*∈*supp*(*A*).

2. Assume to the contrary that *supp*(*A*) is not an LCN-set. Then there exists *A*_1_∈*𝒜*(*supp*(*A*)) with *k*(*A*_1_)<1. It follows by 1 that $v_{h}(A_{1})=1$ for all *h*∈*supp*(*A*_1_) which implies that *A*_1_ | *A*, a contradiction. Thus *supp*(*A*) is an LCN-set and hence min*Δ*(*supp*(*A*))≤|*supp*(*A*)|−2.

3. By Geroldinger and Zhong [14, Lemma 4.2], we have |*G*_0_| = *r*(*G*)+1 and *r*(⟨*G*_0_⟩) = *r*(*G*). If *h*∈⟨*G*_0_∖{*h*}⟩, then *r*(⟨*G*_0_∖{*h*}⟩) = *r*(*G*). Otherwise let *d* = min{*k*∈*ℕ*∣*kh*∈⟨*G*_0_∖*h*⟩} and hence (*G*_0_∖{*h*})∪{*dh*} is also a minimal non-half-factorial LCN-set with $\min\Delta (G_{0})=\max\Delta^{*}(G)$ by Geroldinger and Halter-Koch [7, Lemma 6.7.10]. It follows that *r*(⟨*G*_0_∖{*h*}⟩) = *r*(*G*).

4. Assume to the contrary that there exists *i*∈[1,*k*], say *i* = 1, such that *k*(*U*_1_)<1. Then exp(*G*)*k*(*U*_1_)<exp(*G*). Since $\exp(G)k(U_{1})\in L(U_{1}^{\exp(G)})$, we infer

$$\begin{array}{c} \min L(B^{\exp(G)})\leq \exp(G)k(U_{1})+\exp(G)(k-1)<\exp(G)k\;. \end{array}$$

It follows by max*L*(*B* ^exp(*G*)^)≥exp(*G*)*ℓ* that $\rho (G)=\rho (L(B))\geq \rho (L(B^{\exp(G)}))>\frac{\ell }{k}=\rho (L(B))$, a contradiction.

Assume to the contrary that there exists *j*∈[1,*ℓ*], say *j* = 1, such that *k*(*V*_1_)>1. Then exp(*G*)*k*(*U*_1_)>exp(*G*). Since $\exp(G)k(U_{1})\in L(U_{1}^{\exp(G)})$, we infer

$$\begin{array}{c} \max L(B^{\exp(G)})\geq \exp(G)k(U_{1})+\exp(G)(\ell -1)>\exp(G)\ell \;. \end{array}$$

It follows by min*L*(*B* ^exp(*G*)^)≤exp(*G*)*k* that $\rho (G)=\rho (L(B))\geq \rho (L(B^{\exp(G)}))>\frac{\ell }{k}=\rho (L(B))$, a contradiction.

## 3. The invariants *ρ*(*G*,*d*), ρ*(*G*,*d*), and *K*(*G*,*d*)

First, we introduce new invariants which play a crucial role in the proof of Theorem 1.2 (see Proposition 3.5).

Definition 3.1. Let *d*∈*Δ*_1_(*G*) and *k*∈*ℕ*. We define

$$\begin{array}{c} \rho (G,d,k)=\sup\{\rho (L_{k})|L_{k}\in ℒ(G)\text{ is an AAP with difference }d\text{ and length at least}k\}\geq 1\;. \end{array}$$

Then $(\rho (G,d,k){)}_{k=1}^{\infty }$ is a decreasing sequence of positive real numbers and hence converges. We denote by *ρ*(*G*,*d*) the limit of $(\rho (G,d,k){)}_{k=1}^{\infty }$.

It follows by Geroldinger and Zhong [13, Theorem 3.5] that *ρ*(*G*,1) = *ρ*(*G*) if and only if *G* is not a cyclic group of order 4,6 or 10.

Lemma 3.2. Let *G*_0_⊂*G* be a subset with *Δ*(*G*_0_)≠*∅*. For every *B*∈*ℬ*(*G*_0_) with min*Δ*(*G*_0_)∈*Δ*(*L*(*B*)), we have *ρ*(*G*,min*Δ*(*G*_0_))≥*ρ*(*L*(*B*)).

Proof. Let *B*∈*ℬ*(*G*_0_) with min*Δ*(*G*_0_)∈*Δ*(*L*(*B*)). By definition, *L*(*B*) is an AAP with difference min*Δ*(*G*_0_) and length at least 1. Therefore for every *k*∈*ℕ*, *L*(*B* ^*k*^) is an AAP with difference min*Δ*(*G*_0_) and length at least *k*. Thus for every *k*∈*ℕ*, $\rho (G,\min\Delta (G_{0}),k)\geq \rho (L(B^{k}))\geq \rho (L(B))$ by Lemma 2.2.6. Therefore *ρ*(*G*,min*Δ*(*G*_0_))≥*ρ*(*L*(*B*)).

Definition 3.3. Let *d*∈*Δ*_1_(*G*). We define

$$\begin{array}{ccc} \rho^{*}(G,d) & = & \max\{\rho (G_{0})|G_{0}\subset G\text{ is a non-half-factorial subset with }d\text{ divides }\min\Delta (G_{0})\}\;,\text{ and}\\ K(G,d) & = & \max\{K(G_{0})|G_{0}\subset G\text{ is a non-half-factorial subset with }d\text{ divides }\min\Delta (G_{0})\}\;. \end{array}$$

Note that ρ*(*G*,1) = *ρ*(*G*) and *K*(*G*,1) = *K*(*G*). For every *d*∈*Δ*_1_(*G*), there always exists *G*_0_⊂*G* with *G*_0_ non-half-factorial such that *d* | min*Δ*(*G*_0_) by Lemma 2.5.1. Since *ρ*(*G*_0_)>1 and *K*(*G*_0_)≥1, we have ρ*(*G*,*d*)>1 and *K*(*G*,*d*)≥1. Furthermore, there exist $G_{1},G_{2}\subset G$ with *d* | min*Δ*(*G*_1_) and *d* | min*Δ*(*G*_2_) such that $\rho^{*}(G,d)=\rho (G_{1})$ and *K*(*G*,*d*) = *K*(*G*_2_).

Lemma 3.4. Let *d*∈*Δ*_1_(*G*). Then

1. ρ*(*G*,*d*)≥*ρ*(*G*,*d*).
2. $\rho^{*}(G,d)\leq \rho (G,k_{0}d)$ for some *k*_0_∈*ℕ* with $k_{0}d\in \Delta_{1}(G)$.
3. $\rho^{*}(G,d)=\max\{\rho (G,kd)|k\in \mathbb{N}\text{ and }kd\in \Delta_{1}(G)\}$.

Proof. 1. By the definition of *Δ*_1_(*G*) and *d*∈*Δ*_1_(*G*), for every *k*∈*ℕ*, we let *B*_*k*_∈*ℬ*(*G*) be such that *L*(*B*_*k*_) is an AAP with difference *d* and length at least *k*. Let

$$\begin{array}{c} \ell_{k}=\min\{\min L_{k}|L_{k}\in ℒ(G)\text{ is an AAP with difference }d\text{ and length at least }k\} \end{array}$$

and hence $\underset{k\to \infty }{\lim}\ell_{k}=\infty$ by *ρ*(*G*) is finite.

By Lemma 2.4, there exists a constant *M* such that min*Δ*(*supp*(*B*))∈*Δ*(*L*(*B* ^*M*^)) for all *B*∈*ℬ*(*G*) with *Δ*(*supp*(*B*))≠*∅*. Since $\underset{k\to \infty }{\lim}\ell_{k}=\infty$, we let *k*∈*ℕ* be large enough such that min*L*(*B*_*k*_)≥*M*|*𝒜*(*G*)| and assume

$$\begin{array}{c} B_{k}=U_{1}^{t_{1}}\ldots U_{s}^{t_{s}}=V_{1}\ldots V_{\max L(B_{k})}\;, \end{array}$$

where $s,t_{1},\ldots ,t_{s}\in \mathbb{N}$, $t_{1}+\ldots +t_{s}=\min L(B_{k})$, $U_{1},\ldots ,U_{s}$ are pair-wise distinct atoms, and $V_{1},\ldots ,V_{\max L(B_{k})}$ are atoms.

Since min*L*(*B*_*k*_)≥*M*|*𝒜*(*G*)|, we infer there exists *i*∈[1,*s*], say *i* = 1, such that *t*_1_≥*M*. Set *I* = {*i*∈[1,*s*]∣*t*_*i*_≥*M*} and $G_{0}=supp(\prod_{i\in I}U_{i})$. It follows by the choice of *M* that

$$\begin{array}{c} \min\Delta (G_{0})\in \Delta (L(\underset{i\in I}{\prod }U_{i}^{M}))\;. \end{array}$$

Since *L*(*B*_*k*_) is an AAP with difference *d* and $\prod_{i\in I}U_{i}^{M})$ is a subsequence of *B*_*k*_, we obtain

$$\begin{array}{c} d\;|\;\gcd\Delta (L(B_{k}))\;|\;\gcd\Delta (L(\underset{i\in I}{\prod }\overset{M}{\underset{i}{U}}))\;. \end{array}$$

Therefore *d* | min*Δ*(*G*_0_) and hence $\rho (G_{0})\leq \rho^{*}(G,d)$. Since $\left|\prod_{i\notin I}U_{i}^{t_{i}}|\leq M|𝒜(G)|D(G\right)$, there exists *J*⊂[1,max*L*(*B*_*k*_)] with |*J*|≥max*L*(*B*_*k*_)−*M*|*𝒜*(*G*)|*D*(*G*) such that $\prod_{j\in J}V_{j}$ divides $\prod_{i\in I}U_{i}^{t_{i}}$. It follows by $\min L(\prod_{i\in I}U_{i}^{t_{i}})=\sum_{i\in I}t_{i}$ that

$$\begin{array}{ccc} \max L(B_{k})-M|𝒜(G)|D(G) & \leq & \left|J|\leq \max L(\underset{i\in I}{\prod }\overset{t_{i}}{\underset{i}{U}}\right)\\ \leq & (\underset{i\in I}{\sum }t_{i})\rho (G_{0})\leq \min L(B_{k})\rho^{*}(G,d)\;. \end{array}$$

Therefore

$$\begin{array}{c} \rho (L(B_{k}))\leq \rho^{*}(G,d)+\frac{M|𝒜(G)|D(G)}{\min L(B_{k})}\leq \rho^{*}(G,d)+\frac{M|𝒜(G)|D(G)}{\ell_{k}}\;. \end{array}$$

By definition, we infer $\rho (G,d,k)\leq \rho^{*}(G,d)+\frac{M|𝒜(G)|D(G)}{\ell_{k}}$ which implies that *ρ*(*G*,*d*)≤ρ*(*G*,*d*).

2. Let *G*_0_⊂*G* with *d* | min*Δ*(*G*_0_) and $\rho (G_{0})=\rho^{*}(G,d)$. Then there exists *B*∈*ℬ*(*G*_0_) such that *ρ*(*L*(*B*)) = ρ*(*G*,*d*). Since *supp*(*B*)⊂*G*_0_, we infer min*Δ*(*G*_0_) divides min*Δ*(*supp*(*B*)) and hence *d* divides min*Δ*(*supp*(*B*)).

By Lemma 2.4, there exists a constant *M* such that min*Δ*(*supp*(*B*))∈*Δ*(*L*(*B* ^*M*^)). It follows by Lemma 3.2 and Lemma 2.2.6 that

$$\begin{array}{c} \rho (G,\min\Delta (supp(B)))\geq \rho (L(B^{M}))\geq \rho (L(B))=\rho^{*}(G,d)\;. \end{array}$$

Thus the assertion follows by *d* divides min*Δ*(*supp*(*B*)).

3. For every *k*∈*ℕ* such that *kd*∈*Δ*_1_(*G*), we have $\rho (G,kd)\leq \rho^{*}(G,kd)\leq \rho^{*}(G,d)$ by 1.. Therefore $\rho^{*}(G,d)\geq \max\{\rho (G,kd)|k\in \mathbb{N}\text{ and }kd\in \Delta_{1}(G)\}$. It follows by 2. that $\rho^{*}(G,d)=\max\{\rho (G,kd)|k\in \mathbb{N}\text{ and }kd\in \Delta_{1}(G)\}$.

Proposition 3.5. Suppose *ℒ*(*G*) = *ℒ*(*G* ^*′*^) for some finite abelian group *G* ^*′*^ and let *d*∈*Δ*_1_(*G*). Then $d\in \Delta_{1}(G^{\prime })$, *ρ*(*G*,*d*) = *ρ*(*G* ^*′*^,*d*), and $\rho^{*}(G,d)=\rho^{*}(G^{\prime },d)$.

Proof. Since *ℒ*(*G*) = *ℒ*(*G* ^*′*^), it follows by definition that $\Delta_{1}(G)=\Delta_{1}(G^{\prime })$ and *ρ*(*G*,*kd*) = *ρ*(*G* ^*′*^,*kd*) for every *k*∈*ℕ* such that *kd*∈*Δ*_1_(*G*). By Lemma 3.4.3, we obtain $\rho^{*}(G,d)=\rho^{*}(G^{\prime },d)$.

Lemma 3.6. Let *d*∈*Δ*_1_(*G*). Then ρ*(*G*,*d*)≥*K*(*G*,*d*). In particular, if *d*∈[1,*r*−1], then $\rho^{*}(G,d)\geq K(G,d)\geq 1+\frac{(n_{1}-1)d}{n_{1}}\geq 1+\frac{d}{2}$.

Proof. Suppose *G*_0_⊂*G* with *d* dividing min*Δ*(*G*_0_) and *K*(*G*_0_) = *K*(*G*,*d*). Then there exists *A*∈*𝒜*(*G*_0_) such that *k*(*A*) = *K*(*G*,*d*)≥1. Since

$$\begin{array}{c} \{\exp(G),\exp(G)k(A)\}\subset L(A^{\exp(G)})\;, \end{array}$$

we have

$$\begin{array}{c} \rho^{*}(G,d)\geq \rho (G_{0})\geq \rho (L(A^{\exp(G)}))\geq \frac{\exp(G)k(A)}{\exp(G)}=k(A)=K(G,d)\;. \end{array}$$

In particular, if *d*∈[1,*r*−1], we let $e_{1},\ldots ,e_{d+1}$ be independent elements of order *n*_1_. Set $e_{0}=e_{1}+\ldots +e_{d+1}$ and $G_{0}=\{e_{0},e_{1},\ldots ,e_{d+1}\}$. Then min*Δ*(*G*_0_) = *d* by Lemma 2.3.1.

Since $A=e_{0}\cdot e_{1}^{n_{1}-1}\ldots e_{d+1}^{n_{1}-1}$ is an atom with $k(A)=1+\frac{(n_{1}-1)d}{n_{1}}$, it follows that

$$\begin{array}{c} K(G,d)\geq K(G_{0})\geq k(A)\geq 1+\frac{(n_{1}-1)d}{n_{1}}\geq \frac{1+d}{2}\;. \end{array}$$

Lemma 3.7. Suppose $r\geq \lfloor \frac{n_{r}}{2}\rfloor +1$. Let *G*_0_⊂*G* be a subset with *d* | min*Δ*(*G*_0_), where *d*∈*Δ*_1_(*G*) satisfies $r-1\geq d\geq \lfloor \frac{n_{r}}{2}\rfloor$. If *A*∈*𝒜*(*G*_0_) with *k*(*A*)>1, then $k(A)=1+\frac{sd}{n_{r-d}}$ for some *s*∈*ℕ*.

In particular,

1. $K(G,r-1)=1+\frac{s(r-1)}{n_{1}}$ for some $s\geq n_{1}(u-\sum_{i=1}^{u}\frac{1}{p_{i}^{k_{i}}})$, where $n_{1}=p_{1}^{k_{1}}\ldots p_{u}^{k_{u}}$ with $u,k_{1},\ldots ,k_{u}\in \mathbb{N}$ and $p_{1},\ldots ,p_{u}$ are pairwise distinct primes.
2. if *n*_*r*_ is a prime power, then $K(G,r-1)=1+\frac{\left(n_{1}-1)(r-1\right)}{n_{1}}<r$.
3. if *n*_1_ is not a prime power, then *K*(*G*,*r*−1)>*r*.

Proof. Let *t* = *r*−*d* and we start with the following claim.

Claim A. Let *B*∈*ℬ*(*G*_0_) with $v_{g}(B)\equiv 0\;(\mathrm{mod}\;n_{t})$ for each *g*∈*supp*(*B*) and *supp*(*B*) is an LCN-set. Then there exists $B_{0}\in ℬ(G_{0})$ with *B*_0_ is a product of atoms having cross number 1 and $v_{g}(B_{0})\equiv 0\;(\mathrm{mod}\;n_{t})$ for each *g*∈*supp*(*B*_0_) such that *B* *B*_0_ is a product of atoms having cross number 1.

Proof of Claim A. Assume to the contrary that there exists a *B*∈*ℬ*(*G*_0_) with $v_{g}(B)\equiv 0\;(\mathrm{mod}\;n_{t})$ for each *g*∈*supp*(*B*) and *supp*(*B*) is an LCN-set, such that the assertion does not hold. Suppose |*supp*(*B*)| is minimal in all the counterexamples.

Set *G*_1_ = *supp*(*B*). If for all *g*∈*G*_1_, *ord*(*g*)∣*v*_*g*_(*B*), then *B* is a product of atoms having cross number 1, a contradiction. Therefore there exits $g_{0}\in G_{1}$ such that $ord(g_{0})∤v_{g_{0}}(B)$. Since $v_{g}(B)\equiv 0\;(\mathrm{mod}\;n_{t})$ for each *g*∈*supp*(*B*), we infer $v_{g_{0}}(B)g_{0}\in \langle \{n_{t}g|g\in G_{1}\setminus \{g_{0}\}\}\rangle$ and $n_{t}\neq n_{r}$.

Let $\frac{n_{r}}{n_{t}}=q_{1}^{s_{1}}\ldots q_{v}^{s_{v}}$, where $v,s_{1},\ldots ,s_{v}\in \mathbb{N}$ and $q_{1},\ldots ,q_{v}$ are pairwise distinct primes. Let *i*∈[1,*v*] and $H_{i}=\langle \{\frac{n_{r}}{n_{t}q_{i}^{s_{i}}}n_{t}g|g\in G_{1}\setminus \{g_{0}\}\}\rangle$. Then

$$\begin{array}{c} \frac{n_{r}}{n_{t}q_{i}^{s_{i}}}v_{g_{0}}(B)g_{0}\in H_{i} \end{array}$$

and *H*_*i*_ is an *q*_*i*_-group of rank *r*(*H*_*i*_)≤*r*−*t* = *d* which implies that there exists $E\subset G_{1}\setminus \{g_{0}\}$ with |*E*|≤*r*(*H*_*i*_)≤*d* such that

$$\begin{array}{c} \frac{n_{r}}{n_{t}q_{i}^{s_{i}}}v_{g_{0}}(B)g_{0}\in \langle \left\{\frac{n_{r}}{n_{t}q_{i}^{s_{i}}}n_{t}g|g\in E\right\}\rangle \;. \end{array}$$

Therefore there exists $B_{i}\in ℬ(G_{1})$ such that

$$\begin{array}{c} |supp(B_{i})|\leq d+1,\;v_{g_{0}}(B_{i})=\frac{n_{r}}{n_{t}q_{i}^{s_{i}}}v_{g_{0}}(B),\;\text{ and }n_{t}\;|\;v_{g}(B_{i})\text{ for every }g\in G_{1}\setminus \{g_{0}\}\;. \end{array}$$

Since $supp(B_{i})\subset G_{1}$ is an LCN-set, we have

$$\begin{array}{c} \min\Delta (supp(B_{i}))\leq |supp(B_{i})|-2\leq d-1\;. \end{array}$$

Note that $d\;|\;\min\Delta (G_{0})\;|\;\min\Delta (supp(B_{i}))$. We infer min*Δ*(*supp*(*B*_*i*_)) = 0 and hence *supp*(*B*_*i*_) is half-factorial. Therefore *B*_*i*_ is a product of atoms having cross number 1.

Since $\gcd(\{v_{g_{0}}(B_{i})|i\in [1,v]\})=v_{g_{0}}(B)$, there exist $x_{1},\ldots ,x_{v}\in \mathbb{N}$ such that

$$\begin{array}{c} v_{g_{0}}(BB_{1}^{x_{1}}\ldots B_{v}^{x_{v}})\equiv 0\;(\mathrm{mod}\;ord(g_{0}))\;. \end{array}$$

Therefore $BB_{1}^{x_{1}}\ldots B_{v}^{x_{v}}=g_{0}^{yord(g_{0})}C$ for some *y*∈*ℕ* and $C\in ℬ(G_{1}\setminus \{g_{0}\})$. Note $n_{t}\;|\;v_{g}(B_{i})$ for every *i*∈[1,*v*] and every $g\in G_{1}\setminus \{g_{0}\}$. Thus $n_{t}\;|\;v_{g}(C)$ for each *g*∈*supp*(*C*). Since |*supp*(*C*)|<|*supp*(*B*)|, it follows by the minimality of |*supp*(*B*)| that there exists $C_{0}\in ℬ(supp(G_{0}))$ satisfying *C*_0_ is a product of atoms having cross number 1 and $n_{t}\;|\;v_{g}(C_{0})$ for each *g*∈*supp*(*C*_0_), such that *C* *C*_0_ is a product of atoms having cross number 1. Let $B_{0}=C_{0}B_{1}^{x_{1}}\ldots B_{t_{1}}^{x_{t_{1}}}$. Then *B* *B*_0_ is a product of atoms having cross number 1, a contradiction to our assumption.

Let *A*∈*𝒜*(*G*_0_) be with *k*(*A*)>1. Then Lemma 2.6.2 implies that *supp*(*A*) is an LCN-set. Set $B=A^{n_{t}}$ and hence **Claim A** implies that there exist atoms $W_{1},\ldots ,W_{\ell }\in 𝒜(G_{0})$ having cross number 1, where *ℓ*∈*ℕ*_0_, such that $A^{n_{t}}W_{1}\ldots W_{\ell }$ is a product of atoms having cross number 1. Therefore $\left\{n_{t}k(A)+\ell ,n_{t}+\ell \}\subset L(A^{n_{t}}W_{1}\ldots W_{\ell }\right)$. Since *d* | min*Δ*(*G*_0_), we infer $d\;|\;(n_{t}k(A)-n_{t})$. It follows that $k(A)=1+\frac{sd}{n_{t}}$ for some *s*∈*ℕ*.

Now we begin to prove the “in-particular’’ parts.

1. For every *j*∈[1,*u*] and every *m*∈*ℤ*, we denote by ∥*m*∥_*j*_ the least positive residue of *m* modulo $p_{j}^{k_{j}}$, that is, $∥m{∥}_{j}\in [1,p_{j}^{k_{j}}]$ and $∥m{∥}_{j}\equiv m\;(\mathrm{mod}\;p_{j}^{k_{j}})$.

By definition of *K*(*G*,*r*−1), we have $K(G,r-1)=1+\frac{sd}{n_{1}}$ for some *s*∈*ℕ*. Let *H* be a subgroup of *G* with

$$\begin{array}{c} H≅C_{n_{1}}^{r}≅C_{p_{1}^{k_{1}}}^{r-1}⊕\ldots ⊕C_{p_{u}^{k_{u}}}^{r-1}⊕C_{n_{1}} \end{array}$$

and let $\left(e_{1,1},\ldots ,e_{1,r-1},\ldots ,e_{u,1},\ldots ,e_{u,r-1},e\right)$ be a basis of *H* with *ord*(*e*) = *n*_1_ and $ord(e_{j,i})=p_{j}^{k_{j}}$ for all *i*∈[1,*r*−1], *j*∈[1,*u*]. Set $e_{0}=e+\sum_{j=1}^{u}\sum_{i=1}^{r-1}e_{j,i}$ and $G_{2}=\{e_{1,1},\ldots ,e_{1,r-1},\ldots ,e_{u,1},\ldots ,e_{u,r-1},e,e_{0}\}$. For every *W*∈*𝒜*(*G*_2_), we have

$$\begin{array}{c} W=e_{0}^{v_{e_{0}}(W)}e^{n_{1}-v_{e_{0}}(W)}\underset{i\in [1,r-1],j\in [1,u]}{\prod }e_{j,i}^{p_{j}^{k_{j}}-∥v_{e_{0}}(W){∥}_{j}} \end{array}$$

and

$$\begin{array}{c} k(W)=1+\overset{u}{\underset{j=1}{\sum }}\frac{p_{j}^{k_{j}}-∥v_{e_{0}}(W){∥}_{j}}{p_{j}^{k_{j}}}(r-1)\;. \end{array}$$

We suppose that $U_{1}\ldots U_{\ell_{1}}=V_{1}\ldots V_{\ell_{2}}$ with $\ell_{2}-\ell_{1}=\min\Delta (G_{2})$, where $\ell_{1},\ell_{2}\in \mathbb{N}$ and $U_{1},\ldots ,U_{\ell_{1}}$, $V_{1},\ldots ,V_{\ell_{2}}\in 𝒜(G_{2})$. Then

$$\begin{array}{c} k(U_{1}\ldots U_{\ell_{1}})=\ell_{1}+\overset{\ell_{1}}{\underset{x=1}{\sum }}\overset{u}{\underset{j=1}{\sum }}\frac{p_{j}^{k_{j}}-∥v_{e_{0}}(U_{x}){∥}_{j}}{p_{j}^{k_{j}}}(r-1)=\ell_{2}+\overset{\ell_{2}}{\underset{y=1}{\sum }}\overset{u}{\underset{j=1}{\sum }}\frac{p_{j}^{k_{j}}-∥v_{e_{0}}(V_{y}){∥}_{j}}{p_{j}^{k_{j}}}(r-1)\;. \end{array}$$

Since $v_{e_{0}}(U_{1}\ldots U_{\ell_{1}})=v_{e_{0}}(V_{1}\ldots V_{\ell_{2}})$, we infer

$$\begin{array}{c} \overset{\ell_{1}}{\underset{x=1}{\sum }}∥v_{e_{0}}(U_{x}){∥}_{j}\equiv \overset{\ell_{2}}{\underset{y=1}{\sum }}∥v_{e_{0}}(V_{y}){∥}_{j}\;(\mathrm{mod}\;p_{j}^{k_{j}})\text{ for each }j\in [1,u]\;. \end{array}$$

Therefore

$$\begin{array}{cc} & \overset{\ell_{1}}{\underset{x=1}{\sum }}\frac{p_{j}^{k_{j}}-∥v_{e_{0}}(U_{x}){∥}_{j}}{p_{j}^{k_{j}}}-\overset{\ell_{2}}{\underset{y=1}{\sum }}\frac{p_{j}^{k_{j}}-∥v_{e_{0}}(V_{y}){∥}_{j}}{p_{j}^{k_{j}}}\\ \;=\ell_{1}-\ell_{2}-\frac{\overset{\ell_{1}}{\underset{x=1}{\sum }}∥v_{e_{0}}(U_{x}){∥}_{j}-\overset{\ell_{2}}{\underset{y=1}{\sum }}∥v_{e_{0}}(V_{y}){∥}_{j}}{p_{j}^{k_{j}}}\in \mathbb{Z} \end{array}$$

for each *j*∈[1,*u*], whence

$$\begin{array}{c} \overset{\ell_{1}}{\underset{x=1}{\sum }}\overset{u}{\underset{j=1}{\sum }}\frac{p_{j}^{k_{j}}-∥v_{e_{0}}(U_{x}){∥}_{j}}{p_{j}^{k_{j}}}-\overset{\ell_{2}}{\underset{y=1}{\sum }}\overset{u}{\underset{j=1}{\sum }}\frac{p_{j}^{k_{j}}-∥v_{e_{0}}(V_{y}){∥}_{j}}{p_{j}^{k_{j}}}\in \mathbb{Z}\;. \end{array}$$

Since $\min\Delta (G_{2})=\ell_{2}-\ell_{1}$, we infer *r*−1 | min*Δ*(*G*_2_) which implies that *K*(*G*,*r*−1)≥*K*(*G*_2_). Let $W_{1}\in 𝒜(G_{2})$ be the atom with $v_{e_{0}}(W_{1})=1$. Then $k(W_{1})=1+(u-\sum_{i=1}^{u}\frac{1}{p_{i}^{k_{i}}})(r-1)$.

Since $K(G,r-1)=1+\frac{s(r-1)}{n_{1}}\geq k(W_{1})=1+(u-\sum_{i=1}^{t}\frac{1}{p_{i}^{k_{i}}})(r-1)$ for some *s*∈*ℕ*, it follows that $s\geq n_{1}(u-\sum_{i=1}^{u}\frac{1}{p_{i}^{k_{i}}})$.

2. If *n*_*r*_ is a prime power, then *K*(*G*)<*r* by Lemma 2.1. It follows by 1. that $r>K(G,r-1)=1+\frac{s(r-1)}{n_{1}}\geq 1+\frac{\left(n_{1}-1)(r-1\right)}{n_{1}}$ for some *s*∈*ℕ*. Therefore $K(G,r-1)=1+\frac{\left(n_{1}-1)(r-1\right)}{n_{1}}$.

3. If *n*_1_ is not a prime power, then *u*≥2 and $u-\sum_{i=1}^{u}\frac{1}{p_{i}^{k_{i}}}>u-\frac{u}{2}\geq 1$. It follows by 1. that *K*(*G*,*r*−1)>1+*r*−1 = *r*. □

Proposition 3.8. Let *s*∈*ℕ* be maximal such that *G* has a subgroup isomorphic to $C_{n_{r}}^{s}$. Suppose *d*∈*Δ*_1_(*G*) with $d\geq \max\{\lfloor \frac{n_{r}}{2}\rfloor ,\lfloor \frac{r+1}{2}\rfloor \}$.

1. If *d*≥*r*, then $\rho^{*}(G,d)=\frac{n_{r}}{n_{r}-d}$.
2. If *d*≥*n*_*r*_−1, then ρ*(*G*,*d*) = *K*(*G*,*d*).
3. Suppose *r* = *n*_*r*_−1≥3. If $n_{1}=n_{r}=p^{k}$ for some prime *p* and *k*∈*ℕ*, then $K(G,r-1)<\rho^{*}(G,r-1)=1+\frac{\left(r+1)(r-1\right)}{r+2}<r$. Otherwise, *K*(*G*,*r*−1) = ρ*(*G*,*r*−1).

Proof. 1. Since $d\geq \max\{\lfloor \frac{n_{r}}{2}\rfloor ,\lfloor \frac{r+1}{2}\rfloor \}$ and *d*≥*r*, it follows by Lemma 2.5(items 3 and 4) that *d*>*m*(*G*) and $d\in [n_{r}-s-1,n_{r}-2]$.

Let $e_{1},\ldots ,e_{n_{r}-d-1}$ be independent elements with order *n*_*r*_ and let $e_{0}=-e_{1}-\ldots -e_{n_{r}-d-1}$. Then $\min\Delta (\{e_{0},e_{1},\ldots ,e_{n_{r}-d-1}\})=|n_{r}-(n_{r}-d-1)-1|=d$ by Lemma 2.3.2. Since $A=e_{0}e_{1}\ldots e_{n_{r}-d-1}$ is an atom, we infer $L(A^{n_{r}})=\{n_{r}-d,n_{r}\}$. Therefore

$$\begin{array}{c} \rho^{*}(G,d)\geq \rho (\{e_{0},e_{1},\ldots ,e_{n_{r}-d-1}\})\geq \rho (L(A^{n_{r}}))=\frac{n_{r}}{n_{r}-d}\;. \end{array}$$

Let *G*_0_⊂*G* with *d* | min*Δ*(*G*_0_) be such that $\rho (G_{0})=\rho^{*}(G,d)$. Then there exists *B*∈*ℬ*(*G*_0_) with *ρ*(*L*(*B*)) = ρ*(*G*,*d*). Set

$$\begin{array}{c} B=U_{1}\ldots U_{k}=V_{1}\ldots V_{\ell }, \end{array}$$

where *k* = min*L*(*B*), *ℓ* = max*L*(*B*), and $U_{1},\ldots ,U_{k},V_{1},\ldots ,V_{\ell }\in 𝒜(G_{0})$ are atoms.

If there exists *i*∈[1,*k*] such that *k*(*U*_*i*_)>1, then Lemma 2.6.2 implies that *supp*(*U*_*i*_) is an LCN-set and hence min*Δ*(*supp*(*U*_*i*_))≤*m*(*G*). Since

$$\begin{array}{c} d\;|\;\min\Delta (G_{0})\;|\;\min\Delta (supp(U_{i}))\;, \end{array}$$

we get a contradiction to *d*>*m*(*G*). Therefore *k*(*U*_*i*_)≤1 for all *i*∈[1,*k*].

If there exists *i*∈[1,*ℓ*] such that *k*(*V*_*i*_)<1, then Lemma 2.6.1 implies that $k(V_{i})=\frac{n_{r}-d}{n_{r}}$. Therefore $k(V_{i})\geq \frac{n_{r}-d}{n_{r}}$ for all *i*∈[1,*ℓ*]. It follows that

$$\begin{array}{c} k\geq \overset{k}{\underset{i=1}{\sum }}k(U_{i})=k(B)=\overset{\ell }{\underset{i=1}{\sum }}k(V_{i})\geq \ell \frac{n_{r}-d}{n_{r}}\;. \end{array}$$

Then $\rho^{*}(G,d)=\rho (L(B))=\frac{\ell }{k}\leq \frac{n_{r}}{n_{r}-d}$ and hence $\rho^{*}(G,d)=\frac{n_{r}}{n_{r}-d}$.

2. Let *G*_0_⊂*G* be such that *d* | min*Δ*(*G*_0_) and $\rho (G_{0})=\rho^{*}(G,d)$. If there exists an atom *A*∈*𝒜*(*G*_0_) such that *k*(*A*)<1, then Lemma 2.6.1 implies that $k(A)=\frac{n_{r}-d}{n_{r}}\leq \frac{1}{n_{r}}$, a contradiction to |*A*|≥2. Thus *G*_0_ is an LCN-set. Let *B*∈*ℬ*(*G*_0_) such that $\rho (L(B))=\rho (G_{0})=\rho^{*}(G,d)$. Then

$$\begin{array}{c} \min L(B)K(G,d)\geq k(B)\geq \max L(B) \end{array}$$

which implies that ρ*(*G*,*d*) = *ρ*(*L*(*B*))≤*K*(*G*,*d*).

Let *G*_0_⊂*G* be such that *d* | min*Δ*(*G*_0_) and *K*(*G*_0_) = *K*(*G*,*d*). Then there exists an atom *A*∈*𝒜*(*G*_0_) such that *k*(*A*) = *K*(*G*,*d*)≥1. Since $\left\{n_{r},n_{r}k(A)\}\subset L(A^{n_{r}}\right)$, we infer

$$\begin{array}{c} \rho^{*}(G,d)\geq \rho (G_{0})\geq \rho (L(A^{n_{r}}))\geq k(A)=K(G,d) \end{array}$$

and hence ρ*(*G*,*d*) = *K*(*G*,*d*).

3. Let *r* = *n*_*r*_−1≥3 and we proceed to prove the following claim.

Claim B. Suppose ρ*(*G*,*r*−1)>*K*(*G*,*r*−1) and let *G*_0_⊂*G* be such that (*r*−1) | min*Δ*(*G*_0_) and *ρ*(*G*_0_)>*K*(*G*,*r*−1).

1. There exists *g*∈*G*_0_ with *ord*(*g*) = *n*_*r*_ such that −*g*∈*G*_0_.
2. Let $G_{2}\subset G_{0}\setminus \{g,-g\}$ with |*G*_2_| = *r*. If there exists *a*∈[1,*n*_*r*_−1] such that *ag*∈⟨*G*_2_⟩, then $g=\sum_{h\in G_{2}}h$, $G≅C_{n_{r}}^{r}$, and *G*_2_ is a basis of *G*.
3. $G≅C_{n_{r}}^{r}$, $G_{0}=\{e_{1},\ldots ,e_{r},g,-g\}$, where $g=e_{1}+\ldots +e_{r}$ and $\left(e_{1},\ldots ,e_{r}\right)$ is a basis of *G*, and $\rho (G_{0})=1+\frac{n_{r}(r-1)}{n_{r}+1}$. In particular, $\rho^{*}(G,r-1)=1+\frac{n_{r}(r-1)}{n_{r}+1}$.

Proof of Claim B. By Lemma 2.5.2, we infer that $\min\Delta (G_{0})=r-1=n_{r}-2=\max\Delta^{*}(G)$.

**a.** If *G*_0_ is an LCN-set, then for every *B*∈*ℬ*(*G*_0_), we have

$$\begin{array}{c} \min L(B)K(G,r-1)\geq k(B)\geq \max L(B) \end{array}$$

which implies that *ρ*(*L*(*B*))≤*K*(*G*,*r*−1). Therefore *ρ*(*G*_0_)≤*K*(*G*,*r*−1), a contradiction. Thus there exists *A*∈*𝒜*(*G*_0_) such that *k*(*A*)<1. Lemma 2.6.1 implies that *A* = *g*(−*g*) for some *g*∈*G*_0_ with *ord*(*g*) = *n*_*r*_. Hence {*g*,−*g*}⊂*G*_0_.

**b.** Let *E*⊂*G*_2_ be minimal such that there exists *a*∈[1,*n*_*r*_−1] such that *ag*∈⟨*E*⟩ and let $d_{g}\in [1,n_{r}-1]$ be minimal such that *d*_*g*_ *g*∈⟨*E*⟩. Then there exists an atom *V*∈*𝒜*(*E*∪{*g*}) with $v_{g}(A)=d_{g}$ and |*supp*(*V*)|≤*r*+1. Let $V=g^{d_{g}}T$, where *T*∈*ℱ*(*E*). Then

$$\begin{array}{c} V{\left(-g\right)}^{n_{r}}={\left(g(-g)\right)}^{d_{g}}({\left(-g\right)}^{n_{r}-d_{g}}T)\;,\text{ where }L({\left(-g\right)}^{n_{r}-d_{g}}T)\subset [1,n_{r}-d_{g}]\;. \end{array}$$

Note that for each $\ell \in L({\left(-g\right)}^{n_{r}-d_{g}}T)$, we have *r*−1 | *d*_*g*_+*ℓ*−2. Therefore $L({\left(-g\right)}^{n_{r}-d_{g}}T)=\{n_{r}-d_{g}\}$ or (*d*_*g*_ = 1 and $L({\left(-g\right)}^{n_{r}-1}T)=\{1\}$). We distinguish two cases.

Suppose $L({\left(-g\right)}^{n_{r}-d_{g}}T)=\{n_{r}-d_{g}\}$. Let $T=T_{1}\ldots T_{n_{r}-d_{g}}$ such that (−*g*)*T*_*i*_, $i\in [1,n_{r}-d_{g}]$, are atoms, where $T_{1},\ldots ,T_{n_{r}-d_{g}}\in ℱ(E)$. Thus −*g*∈⟨*E*⟩ which implies that *d*_*g*_ = 1 by the minimality of *d*_*g*_. The minimality of *E* implies that *supp*(*T*_*i*_) = *E* for each *i*∈[1,*n*_*r*_−1]. Then for every *h*∈*E*, $v_{h}(V)\geq n_{r}-1$. Therefore *ord*(*h*) = *n*_*r*_ and $v_{h}(V)=n_{r}-1$. It follows that

$$\begin{array}{c} T_{1}=\ldots =T_{n_{r}-1}=\underset{h\in E}{\prod }h\;. \end{array}$$

If *k*((−*g*)*T*_1_)<1, then Lemma 2.6.1 implies that *T*_1_ = *g*, a contradiction. Therefore

$$\begin{array}{c} k((-g)T_{1})=\frac{1}{n_{r}}+\underset{h\in E}{\sum }\frac{1}{ord(h)}=\frac{1+|E|}{n_{r}}\geq 1\;. \end{array}$$

Since |*E*|≤|*G*_2_|≤*r* and *r* = *n*_*r*_−1, we have |*E*| = |*G*_2_| = *r*. Let $G_{2}=\{e_{1}\ldots ,e_{r}\}$. Then $V=ge_{1}^{n_{r}-1}\ldots e_{r}^{n_{r}-1}$ implies that $\left(e_{1},\ldots ,e_{r}\right)$ is a basis of *G*, $G≅C_{n_{r}}^{r}$, and $g=e_{1}+\ldots +e_{r}$.

Suppose *d*_*g*_ = 1 and $L({\left(-g\right)}^{n_{r}-1}T)=\{1\}$. Note that *V* = *gT* and hence |*T*|≥2. We infer $k({\left(-g\right)}^{n_{r}-1}T)>1$. It follows by Lemma 2.6.2 that {−*g*}∪*E* is an LCN-set and min*Δ*({−*g*}∪*E*)≤|*E*|−1. Since (*r*−1) | min*Δ*({−*g*}∪*E*) and |*E*|≤|*G*_2_| = *r*, we have

$$\begin{array}{c} |E|=|G_{2}|=r\text{ and }\min\Delta (\{-g\}\cup G_{2})=r-1\;. \end{array}$$

Let $E_{1}\subset \{-g\}\cup G_{2}$ be a minimal non-half-factorial LCN-set. Then

$$\begin{array}{c} \min\Delta (E_{1})=\min\Delta (\{-g\}\cup G_{2})=r-1=\max\Delta^{*}(G)\;. \end{array}$$

It follows by Geroldinger and Zhong [14, Lemma 4.2] that |*E*_1_| = *r*+1 and hence $E_{1}=\{-g\}\cup G_{2}$. Therefore

$$\begin{array}{c} \{-g\}\cup G_{2}\text{ is a minimal non-half-factorial LCN-set.} \end{array}$$

Let $G_{2}=\{e_{1},\ldots ,e_{r}\}$ and assume

$$\begin{array}{c} V=ge_{1}^{k_{1}}\ldots e_{r}^{k_{r}},\;\;V_{1}={\left(-g\right)}^{n_{r}-1}e_{1}^{k_{1}}\ldots e_{r}^{k_{r}}\;, \end{array}$$

where $k_{i}\in [1,ord(e_{i})-1]$ for each *i*∈[1,*r*].

If *k*(*V*)>1, then Lemma 2.6.2 and [14, Lemma 4.5] imply

$$\begin{array}{c} \{g\}\cup G_{2}\text{ is a minimal non-half-factorial LCN-set with }\min\Delta (\{g\}\cup G_{2})=\max\Delta^{*}(G)=r-1\;. \end{array}$$

By the minimality of *E* = *G*_2_, we have for every *m*∈[1,*n*_*r*_−1] and every *h*∈*G*_2_, *mg*∉⟨*G*_2_∖{*h*}⟩. Note that *n*_*r*_≥4. Let $I=\{i\in [1,r]|k_{i}\geq \frac{ord(e_{i})}{2}\}$. Then [14, Lemma 4.4.1] implies that

$$\begin{array}{c} W_{1}=g^{2}\underset{i\in I}{\prod }e_{i}^{2k_{i}-ord(e_{i})}\underset{i\in [1,r]\setminus I}{\prod }e_{i}^{2k_{i}} \end{array}$$

and

$$\begin{array}{c} W_{2}={\left(-g\right)}^{n_{r}-2}\underset{i\in I}{\prod }e_{i}^{2k_{i}-ord(e_{i})}\underset{i\in [1,r]\setminus I}{\prod }e_{i}^{2k_{i}} \end{array}$$

are both atoms. Since $V^{2}=\prod_{i\in I}e_{i}^{ord(e_{i})}W_{1}$ and $V_{1}^{2}={\left(-g\right)}^{n_{r}}\prod_{i\in I}e_{i}^{ord(e_{i})}W_{2}$, we infer *r*−1 | |*I*|−1 and *r*−1 | |*I*|. Hence *r* = 2, a contradiction to *r*≥3.

Thus *k*(*V*) = 1 which implies that $k_{1}=\ldots =k_{r}=1$ and $ord(e_{i})=n_{r}$ for each *i*∈[1,*r*]. It follows by *k*(*V*_1_)>1 and [14, Lemma 4.3.2] that $(-g)e_{1}^{n_{r}-1}\ldots e_{r}^{n_{r}-1}$ is also an atom. Therefore $e_{1},\ldots ,e_{r}$ are independent and $g=e_{1}+\ldots +e_{r}$. Since *r*(*G*) = *r* and exp(*G*) = *n*_*r*_, we infer $\left(e_{1},\ldots ,e_{r}\right)$ is a basis of *G* and $G≅C_{n_{r}}^{r}$.

**c.** If for all *W*∈*𝒜*(*G*_0_), *k*(*W*)≤1, then for every *B*∈*ℬ*(*G*_0_), we have

$$\begin{array}{c} \min L(B)\geq k(B)\geq \max L(B)\frac{2}{n_{r}} \end{array}$$

which implies that $\rho (G_{0})\leq \frac{n_{r}}{2}$. It follows by Lemma 3.6 that $K(G,r-1)\geq 1+\frac{r-1}{2}=\frac{n_{r}}{2}$, a contradiction to *ρ*(*G*_0_)>*K*(*G*,*r*−1).

Let *W*∈*𝒜*(*G*_0_) with *k*(*W*)>1. Then *supp*(*W*) is an LCN-set with min*Δ*(*supp*(*W*)) = *r*−1 by Lemma 2.6.2. Let *G*_1_⊂*supp*(*W*) be a minimal non-half-factorial subset. Then min*Δ*(*G*_1_) = *r*−1 and hence |*G*_1_| = *r*+1 by Geroldinger and Zhong [14, Lemma 4.2.1]. Since {*g*,−*g*}⊄*supp*(*W*), we choose *h*∈*G*_1_ such that {*g*,−*g*}∩(*G*_1_∖{*h*}) = *∅*. Lemma 2.6.3 implies *r*(⟨*G*_1_∖{*h*}⟩) = *r*. Thus there exists *a*∈[1,*n*_*r*_−1] such that *ag*∈⟨*G*_1_∖{*h*}⟩. Since |*G*_1_∖{*h*}| = *r*, it follows by **a** and **b** that $G≅C_{n_{r}}^{r}$ and there exists a basis $\left(e_{1},\ldots ,e_{r}\right)$ of *G* such that $g=e_{1}+\ldots +e_{r}$ and $\{e_{1},\ldots ,e_{r},g,-g\}\subset G_{0}$.

Assume to the contrary that there exists $h_{0}\in G_{0}\setminus \{e_{1},\ldots ,e_{r},g,-g\}$. After renumbering if necessary, we may assume that $h_{0}=k_{1}e_{1}+\ldots +k_{t}e_{t}$, where *t*∈[1,*r*], $k_{i}\in [1,n_{r}-1]$ for each *i*∈[1,*t*] and $k_{1}=\min\{k_{1},\ldots k_{t}\}$. Thus $k_{1}g\in \langle \{h_{0},e_{2},\ldots ,e_{r}\}\rangle$. It follows by **b** that $g=h_{0}+e_{2}+\ldots +e_{r}$ and hence $h_{0}=e_{1}$, a contradiction. Therefore $G_{0}=\{e_{1},\ldots ,e_{r},g,-g\}$.

We only need to prove $\rho (G_{0})=1+\frac{n_{r}(r-1)}{n_{r}+1}$ which immediately implies that $\rho^{*}(G,r-1)=1+\frac{n_{r}(r-1)}{n_{r}+1}$.

Since ${\left(ge_{1}^{n_{r}-1}\ldots e_{r}^{n_{r}-1}\right)}^{n_{r}}{\left(-g\right)}^{n_{r}}={\left(g(-g)\right)}^{n_{r}}{\left(e_{1}^{n_{r}}\right)}^{n_{r}-1}\ldots {\left(e_{r}^{n_{r}}\right)}^{n_{r}-1}$, we obtain

$$\begin{array}{c} \rho (G_{0})\geq \frac{n_{r}+r(n_{r}-1)}{n_{r}+1}=1+\frac{n_{r}(r-1)}{n_{r}+1}\;. \end{array}$$

Let *B*∈*ℬ*(*G*_0_) such that *ρ*(*L*(*B*)) = *ρ*(*G*_0_) and assume that

$$\begin{array}{c} B=U_{1}\ldots U_{k}=V_{1}\ldots V_{\ell }\;,\text{ and }\frac{\ell }{k}=\rho (G_{0})\;, \end{array}$$

where *k*,*ℓ*∈*ℕ* and $U_{1},\ldots ,U_{k},V_{1},\ldots ,V_{\ell }$ are atoms.

Note that $𝒜(G_{0})=𝒜(G_{0}\setminus \{-g\})\cup \{g(-g),(-g)e_{1}\ldots e_{r},{\left(-g\right)}^{n_{r}}\}$. By Lemma 2.6.4, we have *k*(*U*_*i*_)≥1 for all *i*∈[1,*k*] and *k*(*V*_*j*_)≤1 for all *j*∈[1,*ℓ*]. Since ${\left((-g)e_{1}\ldots e_{r}\right)}^{n_{r}}={\left(-g\right)}^{n_{r}}\prod_{i\in [1,r]}e_{i}^{n_{r}}$ and $\rho (B^{n_{r}})=\rho (G_{0})$, substituting *B* by $B^{n_{r}}$, if necessary, we can assume that $U_{i}\neq (-g)e_{1}\ldots e_{r}$, $V_{j}\neq (-g)e_{1}\ldots e_{r}$ for each *i*∈[1,*k*] and each *j*∈[1,*ℓ*].

Since there must exist *j*_0_∈[1,*ℓ*] such that $V_{j_{0}}=g(-g)$, there must exists *i*_0_∈[1,*k*] such that $U_{i_{0}}={\left(-g\right)}^{n_{r}}$ which implies that $V_{j}\neq {\left(-g\right)}^{n_{r}}$ for all *j*∈[1,*ℓ*]. If there exists *i*_1_∈[1,*k*] such that $U_{i_{1}}=g^{n_{r}}$, then $\max L(B)=n_{r}+\max L(B{\left(U_{i_{0}}U_{i_{1}}\right)}^{-1})$ which implies that

$$\begin{array}{c} \rho (L(B))=\frac{n_{r}+\max L(B{\left(U_{i_{0}}U_{i_{1}}\right)}^{-1})}{2+\min L(B{\left(U_{i_{0}}U_{i_{1}}\right)}^{-1})}=\rho (G)\;. \end{array}$$

Therefore

$$\begin{array}{c} \rho (L(B))=\frac{n_{r}}{2}=\rho (L(B{\left(U_{i_{0}}U_{i_{1}}\right)}^{-1}))\;. \end{array}$$

It follows by Lemma 3.6 that $K(G,r-1)\geq 1+\frac{r-1}{2}=\frac{n_{r}}{2}$, a contradiction to *ρ*(*G*_0_)>*K*(*G*,*r*−1). If there exists *j*_1_∈[1,*ℓ*] such that $V_{j_{1}}=g^{n_{r}}$, then $\max L(B{\left(-g\right)}^{n_{r}})=n_{r}+\ell -1$ which implies that

$$\begin{array}{c} \rho (L(B{\left(-g\right)}^{n_{r}}))\geq \frac{n_{r}+\ell -1}{k}>\frac{\ell }{k}=\rho (G_{0})\;, \end{array}$$

a contradiction.

To sum up, we obtain

$$\begin{array}{c} \left\{U_{i}|i\in [1,k]\}\subset (A(G_{0}\setminus \{-g\})\setminus \{g^{n_{r}}\})\cup \{{\left(-g\right)}^{n_{r}}\right\} \end{array}$$

and

$$\begin{array}{c} \{V_{j}|j\in [1,\ell ]\}\subset 𝒜(G_{0}\setminus \{g,-g\})\cup \{(-g)g\}\;. \end{array}$$

Let $I_{1}=\{i\in [1,k]|U_{i}={\left(-g\right)}^{n_{r}}\}$, $I_{2}=\{i\in [1,k]|v_{g}(U_{i})\geq 1\}$, and *J* = {*j*∈[1,*ℓ*]∣*V*_*j*_ = *g*(−*g*)}. Then

$$\begin{array}{c} n_{r}|I_{1}|=|J|=v_{-g}(B)=v_{g}(B)=\underset{i\in I_{2}}{\sum }v_{g}(U_{i})\geq |I_{2}| \end{array}$$

and

$$\begin{array}{ccc} \frac{2}{n_{r}}|J|+\ell -|J|=k(B) & = & \underset{i\in I_{2}}{\sum }k(U_{i})+k-|I_{2}|\\ = & \underset{i\in I_{2}}{\sum }(1+\frac{n_{r}-v_{g}(U_{i})}{n_{r}}(n_{r}-2))+k-|I_{2}|\\ = & k+(n_{r}-2)|I_{2}|-\frac{n_{r}-2}{n_{r}}\underset{i\in I_{2}}{\sum }v_{g}(U_{i})\\ = & k+(n_{r}-2)|I_{2}|-\frac{\left|J|(n_{r}-2\right)}{n_{r}}\;. \end{array}$$

Therefore $k\geq |I_{1}|+|I_{2}|\geq \frac{n_{r}+1}{n_{r}}|I_{2}|$ and $\frac{\ell }{k}=1+\frac{\left|I_{2}\right|}{k}(n_{r}-2)\leq 1+\frac{n_{r}(n_{r}-2)}{n_{r}+1}$. It follows that $\rho (G_{0})=1+\frac{n_{r}(r-1)}{n_{r}+1}$.

We distinguish two cases to finish the proof.

Suppose that $G≅C_{p^{k}}^{p^{k}-1}$ for some prime *p* and *k*∈*ℕ* with *p* ^*k*^≥4. Then $K(G,p^{k}-2)=1+\frac{\left(p^{k}-1)(p^{k}-2\right)}{p^{k}}$ by Lemma 3.7.2. Let $\left(e_{1},\ldots ,e_{p^{k}-1}\right)$ be a basis of *G* and $G_{0}=\{e_{1},\ldots ,e_{p^{k}-1},g,-g\}$, where $g=\sum_{i\in [1,p^{k}-1]}e_{i}$. By Lemma 2.3.3, we have $p^{k}-2=\min\Delta (G_{0})$. Since

$$\begin{array}{c} {\left(ge_{1}^{p^{k}-1}\ldots e_{r}^{p^{k}-1}\right)}^{p^{k}}{\left(-g\right)}^{p^{k}}={\left(g(-g)\right)}^{p^{k}}{\left(e_{1}^{p^{k}}\right)}^{p^{k}-1}\ldots {\left(e_{r}^{p^{k}}\right)}^{p^{k}-1}\;, \end{array}$$

we obtain

$$\begin{array}{c} \rho^{*}(G,p^{k}-2)\geq \rho (G_{0})\geq \frac{p^{k}+(p^{k}-1)r}{p^{k}+1}=1+\frac{p^{k}(p^{k}-2)}{p^{k}+1}>K(G,p^{k}-2)\;. \end{array}$$

It follows by **Claim B** that

$$\begin{array}{c} K(G,p^{k}-2)<\rho^{*}(G,p^{k}-2)=1+\frac{p^{k}(p^{k}-2)}{p^{k}+1}<p^{k}-1\;. \end{array}$$

Suppose that $G≇C_{p^{k}}^{p^{k}-1}$ for any prime *p* and any *k*∈*ℕ*. Assume to the contrary that *K*(*G*,*r*−1)<ρ*(*G*,*r*−1). Then **Claim B** implies that $G≅C_{n_{r}}^{r}$ and *ρ*(*G*,*r*−1)<*r*. Since *n*_*r*_ is not prime power, it follows by Lemma 3.7.3 that *K*(*G*,*r*−1)>*r*, a contradiction. □

## 4. Proof of main theorems

From now on, we assume *G* ^*′*^ is a further finite abelian group with $G^{\prime }≅C_{n_{1}^{\prime }}⊕\ldots ⊕C_{n_{r}^{\prime }}\prime$, where $r^{\prime },n_{1}^{\prime },\ldots ,nr^{\prime }\prime \in \mathbb{N}$ and $1<n_{1}^{\prime }\;|\;\ldots \;|\;n_{r}^{\prime }\prime$. For convenience, we collect some necessary results which will be used all through the following two sections without further mention.

Lemma 4.1. Suppose *ℒ*(*G*) = *ℒ*(*G* ^*′*^) and *d*∈*Δ*_1_(*G*). Then

1. If *G* is isomorphic to a subgroup of *G* ^*′*^, then *G*≅*G* ^*′*^.
2. $\max\{r(G)-1,\exp(G)-2\}=\max\{r(G^{\prime })-1,\exp(G^{\prime })-2\}$.
3. $d\in \Delta_{1}(G^{\prime })$ and $\rho^{*}(G,d)=\rho^{*}(G^{\prime },d)$.

Proof. 1. By Geroldinger and Halter-Koch [7, Proposition 7.3.1.3], we have *D*(*G*) = *D*(*G* ^*′*^). It follows from [7, Proposition 5.1.3.2 and 5.1.11.1] that *G*≅*G* ^*′*^.

2. follows from [7, Corollary 4.3.16] and [14, Theorem 1.1.3].

3. See Proposition 3.5.

Theorem 4.2. Suppose *ℒ*(*G*) = *ℒ*(*G* ^*′*^). Then

1. If *r*≥*n*_*r*_−1 and *n*_1_≠2, then $r(G)=r(G^{\prime })\geq \exp(G^{\prime })-1$.
2. If *r*≥*n*_*r*_−1, *n*_1_≠2, and *n*_*r*_ is a prime power , then *r*(*G*) = *r*(*G* ^*′*^) and $n_{1}=n_{1}^{\prime }$.
3. If *r*≤*n*_*r*_−3, then exp(*G*) = exp(*G* ^*′*^) and $r(G^{\prime })\leq n_{r}-3$.
4. If $\lfloor \frac{n_{r}}{2}\rfloor +1\leq r\leq n_{r}-3$, then exp(*G*) = exp(*G* ^*′*^) and *r*(*G*) = *r*(*G* ^*′*^).
5. If *r*≤*n*_*r*_−3 and Δ*(*G*) is an interval, then exp(*G*) = exp(*G* ^*′*^) and *r*(*G*) = *r*(*G* ^*′*^).

Proof. 1. Assume to the contrary that *r*(*G* ^*′*^)≠*r*(*G*) = *r*. Since $r-1=\max\{r(G)-1,\exp(G)-2\}=\max\{r(G^{\prime })-1,\exp(G^{\prime })-2\}$, it follows that $r(G^{\prime })-1<\exp(G^{\prime })-2=r-1$. Let *d* = *r*−1. Then $d\in \Delta_{1}(G)=\Delta_{1}(G^{\prime })$. Since $\rho^{*}(G,d)\geq 1+\frac{(n_{1}-1)d}{n_{1}}$ by Lemma 3.6 and $\rho^{*}(G^{\prime },d)=\frac{\exp(G^{\prime })}{2}$ by Proposition 3.8.1, it follows that $\rho^{*}(G^{\prime },d)=\frac{\exp(G^{\prime })}{2}=\rho^{*}(G,d)\geq 1+\frac{\left(n_{1}-1)(\exp(G^{\prime })-2\right)}{n_{1}}$, a contradiction to *n*_1_≠2. Thus $r-1=r(G^{\prime })-1=\max\{r(G^{\prime })-1,\exp(G^{\prime })-2\}$ and hence $r(G^{\prime })\geq \exp(G^{\prime })-1$.

2. Note that *r*≥*n*_*r*_−1≥2. If *r* = 2, then $G≅C_{3}⊕C_{3}$. Therefore *D*(*G*) = *D*(*G* ^*′*^) = 5 and $\max\{3-2,2-1\}=\max\{\exp(G^{\prime })-2,r(G^{\prime })-1\}$. It follows that *G*≅*G* ^*′*^. Thus we can assume *r*≥3.

By 1., we have $r(G)=r(G^{\prime })\geq \exp(G^{\prime })-1$. Since *n*_*r*_ is a prime power and *n*_*r*_≥3, it follows by Lemma 3.7.2 that $K(G,r-1)=1+\frac{n_{r}(r-1)}{n_{r}}<r$. Then *r*≥3 and Propositions 3.8.2 and 3.8.2 imply that ρ*(*G*,*r*−1)<*r* and hence $\rho^{*}(G^{\prime },r-1)=\rho^{*}(G,r-1)<r$. Therefore

$$\begin{array}{c} 1+\frac{\left(n_{1}^{\prime }-1)(r-1\right)}{n_{1}^{\prime }}\leq K(G^{\prime },r-1)\leq \rho^{*}(G^{\prime },r-1)<r \end{array}$$

by Lemma 3.6. It follows by Lemma 3.7.1 that $K(G^{\prime },r-1)=1+\frac{\left(n_{1}^{\prime }-1)(r-1\right)}{n_{1}^{\prime }}$.

If *K*(*G*,*r*−1) = ρ*(*G*,*r*−1) and $K(G^{\prime },r-1)=\rho^{*}(G^{\prime },r-1)$, then $1+\frac{\left(n_{r}-1)(r-1\right)}{n_{r}}=1+\frac{\left(n_{1}^{\prime }-1)(r-1\right)}{n_{1}^{\prime }}$ which infers $n_{1}=n_{r}=n_{1}^{\prime }$.

If *K*(*G*,*r*−1)<ρ*(*G*,*r*−1) and $K(G^{\prime },r-1)=\rho^{*}(G^{\prime },r-1)$, then $G≅C_{n_{r}}^{r}$, *r* = *n*_*r*_−1, and $\rho^{*}(G,r-1)=1+\frac{\left(n_{r})(r-1\right)}{n_{r}+1}$ by Proposition 3.8.3. Therefore $1+\frac{\left(n_{r})(r-1\right)}{n_{r}+1}=1+\frac{\left(n_{1}^{\prime }-1)(r-1\right)}{n_{1}^{\prime }}$ which infers $r+2=n_{r}+1=n_{1}^{\prime }\leq \exp(G^{\prime })$, a contradiction.

If *K*(*G*,*r*−1) = ρ*(*G*,*r*−1) and $K(G^{\prime },r-1)<\rho^{*}(G^{\prime },r-1)$, then $G^{\prime }≅C_{\exp(G^{\prime })}^{r}$, *r* = exp(*G* ^*′*^)−1, and $\rho^{*}(G^{\prime },r-1)=1+\frac{\exp(G^{\prime })(r-1)}{\exp(G^{\prime })+1}$ by Proposition 3.8.3. Therefore $1+\frac{\left(n_{1}-1)(r-1\right)}{n_{1}}=1+\frac{\left(\exp(G^{\prime }))(r-1\right)}{\exp(G^{\prime })+1}$ which infers $n_{r}\geq n_{1}=\exp(G^{\prime })+1=r+2$, a contradiction.

If *K*(*G*,*r*−1)<ρ*(*G*,*r*−1) and $K(G^{\prime },r-1)<\rho^{*}(G^{\prime },r-1)$, then $G≅C_{r+1}^{r}≅G^{\prime }$ by Proposition 3.8.3.

3. Note that *n*_*r*_≥4. Assume to the contrary that $r(G^{\prime })\geq n_{r}-2$. Let $d=n_{r}-3\in [1,r(G^{\prime })-1]$. Therefore $d\in \Delta_{1}(G^{\prime })=\Delta_{1}(G)$ and Lemma 3.6 implies that $\rho^{*}(G^{\prime },d)\geq 1+\frac{d}{2}$. Since *d*≥max{*r*,⌊*n*_*r*_∕2⌋}, Proposition 3.8.1 implies that $\rho^{*}(G,d)=n_{r}∕3<1+\frac{d}{2}$, a contradiction to $\rho^{*}(G,d)=\rho^{*}(G^{\prime },d)$.

Thus $r(G^{\prime })\leq n_{r}-3$. Since $n_{r}-2=\max\{r(G)-1,\exp(G)-2\}=\max\{r(G^{\prime })-1,\exp(G^{\prime })-2\}$, it follows that $n_{r}-2=\exp(G^{\prime })-2$.

4. By 3, exp(*G*) = exp(*G* ^*′*^) and $r(G^{\prime })\leq \exp(G^{\prime })-3$. Assume to the contrary that *r*(*G*)≠*r*(*G* ^*′*^).

Suppose that *r*(*G*)>*r*(*G* ^*′*^). Choose *d* = *r*−1. Therefore $d\in \Delta_{1}(G)=\Delta_{1}(G^{\prime })$ and Lemma 3.6 implies that $\rho (G,d)\geq 1+\frac{r-1}{2}$. Since $d\geq \max\{r(G^{\prime }),\lfloor \exp(G^{\prime })∕2\rfloor \}$, Proposition 3.8.1 implies that $\rho (G^{\prime },d)=\frac{\exp(G^{\prime })}{\exp(G^{\prime })-d}\leq \frac{n_{r}}{4}<1+\frac{r-1}{2}$, a contradiction to $\rho^{*}(G,d)=\rho^{*}(G^{\prime },d)$.

Suppose that *r*(*G*)<*r*(*G* ^*′*^). Choose *d* = *r*∈[1,*r*(*G* ^*′*^)−1]. Then $d\in \Delta_{1}(G^{\prime })=\Delta_{1}(G)$ and Lemma 3.6 implies that $\rho^{*}(G^{\prime },d)\geq 1+\frac{r}{2}$. Since *d*≥max{*r*,⌊*n*_*r*_∕2⌋}, Proposition 3.8.1 implies that $\rho^{*}(G,d)=\frac{n_{r}}{n_{r}-r}=1+\frac{r}{n_{r}-r}<1+\frac{r}{2}$, a contradiction to $\rho^{*}(G,d)=\rho^{*}(G^{\prime },d)$.

5. By 3, we have $r(G^{\prime })+3\leq \exp(G^{\prime })=n_{r}$ and by 4, we can assume that $r\leq \lfloor \frac{n_{r}}{2}\rfloor$ and $r(G^{\prime })\leq \lfloor \frac{n_{r}}{2}\rfloor$. Since Δ*(*G*) is an interval, we obtain that $\Delta^{*}(G^{\prime })=\Delta_{1}(G^{\prime })=\Delta_{1}(G)=\Delta^{*}(G)$ is an interval by Lemma 2.5.5. Let *k* be maximal such that there exists a subgroup *H* of *G* with $H≅C_{n_{r}}^{k}$ and let *k* ^*′*^ be maximal such that there exists a subgroup *H* ^*′*^ of *G* ^*′*^ with $H^{\prime }≅C_{n_{r}}^{k^{\prime }}$. Then 2*r*≥*r*+*k*≥*n*_*r*_−2 and $2r(G^{\prime })\geq r(G^{\prime })+k^{\prime }\geq n_{r}-2$ by Lemma 2.5.5.

Assume to the contrary that *r*≠*r*(*G* ^*′*^) and by symmetry, we can assume *r*<*r*(*G* ^*′*^). Thus $\frac{n_{r}}{2}-1\leq r<r(G^{\prime })\leq \lfloor \frac{n_{r}}{2}\rfloor$ which implies that *n*_*r*_ is even, *n*_*r*_ = 2*r*+2, and *r*(*G* ^*′*^) = *r*+1. Since Δ*(*G*) and $\Delta^{*}(G^{\prime })$ are intervals, it follows by Lemma 2.5.5 that $G≅C_{n_{r}}^{r}$ and *k* ^*′*^≥*r*. Then *G* is a subgroup of *G* ^*′*^ which implies that *G*≅*G* ^*′*^ by Lemma 4.1.1, a contradiction.

Proof of Theorem 1.2. By definition of transfer Krull monoids, it follows that $ℒ(G)=ℒ(H)=ℒ(H^{\prime })=ℒ(G^{\prime })$.

1. Let *r*≤*n*−3. If Δ*(*G*) is not an interval, then [27, Theorems 1.1 and 1.2] implies that *G*≅*G* ^*′*^.

Suppose Δ*(*G*) is an interval. Then Theorem 4.2.5 implies that *n* = exp(*G* ^*′*^) and *r* = *r*(*G* ^*′*^). Therefore *G* ^*′*^ is isomorphic to a subgroup of *G* which implies that *G*≅*G* ^*′*^ by Lemma 4.1.1.

2. If *n* = 2, then *G* is an elementary two-group and the assertion follows by Geroldinger and Halter-Koch [7, Theorem 7.3.3]. We assume *n*≥3 is a prime power. Then Theorem 4.2.2 implies that *r* = *r*(*G* ^*′*^) and $n=n_{1}^{\prime }$. Therefore *G* is isomorphic to a subgroup of *G* ^*′*^ which implies that *G*≅*G* ^*′*^ by Lemma 4.1.1.

## 5. Concluding remarks and conjectures

Throughout this section, let $G≅C_{n}^{r}$ with *D*(*G*)≥4 and $n=p_{1}^{k_{1}}\ldots p_{u}^{k_{u}}$, where $u,k_{1},\ldots ,k_{u}\in \mathbb{N}$ and $p_{1},\ldots ,p_{u}$ are pair-wise distinct primes.

Conjecture 5.1. Suppose *r*≥*n*−1. Then the following hold:

1. $K(G,r-1)=1+\frac{s(r-1)}{n}$ for some *s*∈*ℕ* with gcd(*s*,*n*) = 1.
2. $K(G,r-1)=1+(\sum_{i=1}^{u}\frac{p_{i}^{k_{i}}-1}{p_{i}^{k_{i}}})(r-1)$.

It is easy to see that *C*2 implies *C*1. By Lemma 3.7.1, we have $K(G,r-1)=1+\frac{s(r-1)}{n}$ for some *s*∈*ℕ* and if *n* is a prime power, then *C*2 holds.

Proposition 5.2. Let *G* ^*′*^ be a finite abelian group with *ℒ*(*G* ^*′*^) = *ℒ*(*G*). If *r*≥*n*−1 and **C1** holds, then *G*≅*G* ^*′*^.

Proof. If *n* is a prime power, then the assertion follows by Theorem 1.2.

Suppose *n* is not a prime power. Then *r*≥*n*−1≥5. Let *ℒ*(*G*) = *ℒ*(*G* ^*′*^) and let $G^{\prime }≅C_{n_{1}^{\prime }}⊕\ldots ⊕C_{n_{r}^{\prime }}\prime$ with $r^{\prime },n_{1}^{\prime },\ldots ,n_{r}^{\prime }\prime \in \mathbb{N}$ and $1<n_{1}^{\prime }\;|\;\ldots \;|\;n_{r}^{\prime }\prime$. Then Theorem 4.2.1 implies that $r=r^{\prime }\geq n_{r}^{\prime }\prime -1$. By Lemma 3.7.1 and Proposition 3.8(items 2. and 3.), we have $\rho^{*}(G,r-1)=K(G,r-1)=1+\frac{s(r-1)}{n}$ for some *s*∈*ℕ*. If $\rho^{*}(G^{\prime },r-1)=K(G^{\prime },r-1)$, then Lemma 3.7.1 implies that $\rho^{*}(G^{\prime },r-1)=1+\frac{s^{\prime }(r-1)}{n_{1}^{\prime }}$ for some *s* ^*′*^∈*ℕ*. Thus $\rho^{*}(G,r-1)=\rho^{*}(G^{\prime },r-1)$ implies that $\frac{s}{n}=\frac{s^{\prime }}{n_{1}^{\prime }}$ and hence $n\;|\;n_{1}^{\prime }$ by gcd(*s*,*n*) = 1. Therefore *G* is isomorphic to a subgroup of *G* ^*′*^ which implies that *G*≅*G* ^*′*^ by Lemma 4.1.1.

Suppose $\rho^{*}(G^{\prime },r-1)\neq K(G^{\prime },r-1)$. Then Lemma 3.6 implies that $\rho^{*}(G^{\prime },r-1)>K(G^{\prime },r-1)$ and hence Proposition 3.8(items 2. and 3.) implies that $n_{r}^{\prime }=r+1$ and $\rho^{*}(G,r-1)=1+\frac{\left(r+1)(r-1\right)}{r+2}$. Thus $\rho^{*}(G,r-1)=\rho^{*}(G^{\prime },r-1)$ implies that $\frac{s}{n}=\frac{r+1}{r+2}$. Since gcd(*s*,*n*) = 1, we have *n* = *r*+2, a contradiction.

Recall that $K^{*}(G^{\prime })\leq K(G^{\prime })$ for all finite abelian group *G* ^*′*^ and there is known no group *G* ^*′*^ with $K^{*}(G^{\prime })<K(G^{\prime })$.

Proposition 5.3. Let *G* ^*′*^ be a finite abelian group with *ℒ*(*G* ^*′*^) = *ℒ*(*G*). If *r*≥max{(*u*−1)*n*+1,*n*}≥3 and *K*(*G*) = K*(*G*), then *G*≅*G* ^*′*^.

Proof. If *u* = 1, then the assertion follows by Theorem 1.2.2. Suppose *u*≥2. Note $K(G)=K^{*}(G)=\frac{1}{n}+r(\sum_{i=1}^{u}\frac{p_{i}^{k_{i}}-1}{p_{i}^{k_{i}}})=1+\frac{s}{n}(r-1)+\sum_{i=1}^{u}\frac{p_{i}^{k_{i}}-1}{p_{i}^{k_{i}}}-\frac{n-1}{n}$, where $s=n(\sum_{i=1}^{u}\frac{p_{i}^{k_{i}}-1}{p_{i}^{k_{i}}})$. Since *r*≥(*ω*(*n*)−1)*n*+1, we have $\sum_{i=1}^{u}\frac{p_{i}^{k_{i}}-1}{p_{i}^{k_{i}}}-\frac{n-1}{n}<u-1\leq \frac{r-1}{n}$. It follows by Lemma 3.7.1 that $1+\frac{s}{n}(r-1)\leq K(G,r-1)=1+\frac{s^{\prime }}{n}(r-1)\leq 1+\frac{s+1}{n}(r-1)$, where *s* ^*′*^∈*ℕ*. Therefore $1+\frac{s}{n}(r-1)=K(G,r-1)$. Since $s=n(\sum_{i=1}^{u}\frac{p_{i}^{k_{i}}-1}{p_{i}^{k_{i}}})$, we have gcd(*s*,*n*) = 1. The assertion follows by Proposition 5.2.
